# Microbial community assembly and functional profiles along the soil-root continuum of salt-tolerant *Suaeda glauca* and *Suaeda salsa*

**DOI:** 10.3389/fpls.2023.1301117

**Published:** 2023-11-17

**Authors:** Luyao Tang, Le Zhan, Yanan Han, Zhengran Wang, Lei Dong, Zhong Zhang

**Affiliations:** ^1^ School of Pharmacy, Weifang Medical University, Weifang, Shandong, China; ^2^ Key Laboratory of Biological Medicines in Universities of Shandong Province, Weifang Key Laboratory of Antibody Medicines, School of Life Science and Technology, Weifang Medical University, Weifang, Shandong, China

**Keywords:** community assembly, functional profiles, microbiomes, salt stress, *Suaeda*

## Abstract

Developing and planting salt-tolerant plants has become a promising way to utilize saline-alkali land resources and ensure food security. Root-associated microbes of salt-tolerant plants have been shown to promote plant growth and alleviate high salt stress, yet very little is known about the salt resistance mechanisms of core microbes in different niches. This study characterized the microbial community structures, assembly processes, and functional profiles in four root-related compartments of two salt-tolerant plants by amplicon and shotgun metagenomic sequencing. The results showed that both plants significantly altered the microbial community structure of saline soils, with greater microbial alpha diversity in the rhizosphere or rhizoplane compared with bulk soils. Stochastic process dominated the microbial assembly processes, and the impact was stronger in *Suaeda salsa* than in *S. glauca*, indicating that *S. salsa* may have stronger resistance abilities to changing soil properties. Keystone species, such as *Pseudomonas* in the endosphere of *S. glauca* and *Sphingomonas* in the endosphere of *S. salsa*, which may play key roles in helping plants alleviate salt stress, were identified by using microbial co-occurrence network analysis. Furthermore, the microbiomes in the rhizoplane soils had more abundant genes involved in promoting growth of plants and defending against salt stress than those in bulk soils, especially in salt-tolerant *S. salsa*. Moreover, microbes in the rhizoplane of *S. salsa* exhibited higher functional diversities, with notable enrichment of genes involved in carbon fixation, dissimilar nitrate reduction to ammonium, and sulfite oxidation. These findings revealed differences and similarities in the microbial community assembly, functional profiles and keystone species closely related to salt alleviation of the two salt-tolerant plants. Overall, our study provides new insights into the ecological functions and varied strategies of rhizosphere microbes in different plants under salt stress and highlights the potential use of keystone microbes for enhancing salt resistance of plants.

## Introduction

Soil salinization is not only a worldwide environmental problem but also one of the most important problems restricting agricultural production, food security, and sustainability. The total area of global saline-alkali land is 954.38 million hectares, of which China contains 99.13 million hectares, accounting for more than one-tenth of the total area in the world (Yue et al., 2020). An approximately 0.6 million hectares in Shandong Province are saline-alkali land area, accounting for 3.8% of the land area. High salt and scarce water are limiting factors for the development of saline-alkali land. The salinization degree of saline-alkali soil in coastal areas of Weifang City is relatively high, with a total salt content of >0.8% and a pH >8.2 (Wang et al., 2022b; Kang, 2023). At present, many strategies (including physical, chemical, and biological) have been developed to improve saline-alkali soil (Meena et al., 2019). Compared to other methods, bioremediation technology is an efficient, sustainable, and environmentally friendly soil remediation method. For example, approximately 100 hectares of salt-tolerant *Tamarix* were planted in coastal areas to reduce the degree of soil salinization (Wang et al., 2022a).

Planting salt-tolerant plants can not only effectively reduce the salinity but also alter various other variety properties of soil (Moreira et al., 2020). In this process, the microbial communities and plants living in different spatial niches have formed complex and dynamic interactions in the long-term co-evolution process (Liu et al., 2020a). The rhizosphere microbial communities showed unique advantages in enhancing the resistance ability of the host to salt stress. Furthermore, plants provide rich nutrients for microorganisms through photosynthesis, affecting microbial community compositions in root-related niches. Studies have shown that salt-tolerant plants have special root-associated microbiomes (Mishra et al., 2018). The regulation of plant growth by microorganisms can be achieved through direct ways to enhance biological enzymes (such as ACC deaminase) and produce plant hormone signals (such as abscisic acid) (Etesami and Beattie, 2018) or through indirect ways to alleviate osmotic stress and accumulate antioxidant compounds (Guo et al., 2021). However, the mechanisms of root-associated microorganisms in promoting plant growth are usually conducted in specific microbial species, and the role of the whole microbial community in salt-tolerant plants is lacking.

Plants of the genus *Suaeda* have strong saline-alkali tolerance and are currently pioneer plants for improving and greening saline-alkali soil. *Suaeda* plants contain a variety of rich nutrients and medicinal ingredients (Xu et al., 2023) and have been widely studied in the fields of medicine and food. Many studies have reported the salt tolerance mechanisms of plants belonging to the genus *Suaeda*, which provides a theoretical foundation for better use of saline-alkali land (Wang et al., 2021a; Yu et al., 2022). *Suaeda* has become an important potential plant for soil remediation because of its strong phytoextraction ability to enhance tolerance to saline-alkali and heavy metals (Joshi et al., 2020; Li et al., 2022). Additionally, it can significantly adjust osmo-protective compounds such as soluble sugars and antioxidant systems to resist high salinity (Joshi et al., 2023). Apart from the endogenous mechanism of plant resistance, the microbiota has been suggested to be capable of reducing the effect of high salt on the growth of the *Suaeda* plants and promoting their adaptation to salinity. *Arbuscular mycorrhizal* (AM) fungi regulate the ion balance and influence the bacterial community composition of the rhizosphere, enriching rhizosphere bacteria to help plant growth (Diao et al., 2021a). AM fungi further regulate salt tolerance-related genes and metabolic pathways to promote the adaptation of *S. salsa* to saline environments (Diao et al., 2021b). *Providencia vermicola* BR68 and *Sarocladium kiliense* FS18 isolated from *S. salsa* not only dramatically improved net photosynthetic rates and antioxidant enzyme activities, but also notably reduced the proline contents and production rates of reactive oxygen species, which ultimately improved the physiology and promoted the growth of maize under salt stress (Wang et al., 2022c). Moreover, inoculating bacteria with the two endophytes *Sphingomonas prati* and *Sphingomonas zeicaulis* improved intracellular osmotic metabolism and catalase production in antioxidant enzyme systems of *S. salsa*, thus significantly improving the resistance abilities of salt tolerance of plants (Guo et al., 2021). In recent decades, shotgun metagenomic sequencing has been widely applied to study the soil and rhizosphere microbiome of plants, which provides taxonomic, genomic, and functional profiles for a specific community (Xu et al., 2018). In contrast, there are few reports on the microbial assembly and functional profiles in different niches of the two salt-tolerant *Suaeda* species.

Our work aimed to identify the distinctions in root-related microbial community assembly and functional profiles of two salt-tolerant plants. Bulk soil, rhizosphere soil, rhizoplane soil, and roots of two plants (*S. glauca*, *S. salsa*) were sampled, and changes in microbial community compositions along soil-root continuums were investigated by performing amplicon sequencing. Shotgun metagenomic studies were also conducted to reveal the microbiome functional profiles in different niches between the two plants. In addition, we explored the dominant microbial populations of two salt-tolerant plants and assessed the ecological function of their microbial communities. This study provided a detailed understanding of root-related microbial communities and functional characteristics and revealed various salt stress tolerance mechanisms of different salt-tolerant plants.

## Materials and methods

### Sample collection

Samples were collected from saline-alkaline land (37°5′54.32″N, 119°2′44.10″E) in coastal areas of Weifang City, China, on October 22nd, 2022. Soil and root samples were collected according to four root-related compartments, including bulk soil, rhizosphere soil, rhizoplane soil and endosphere. Specifically, bulk soil was sampled at a distance of 20 cm away from the root after removing the top 1~2 cm soil layer. The other root-associated samples were collected as previously described (Zheng et al., 2021). A total of 48 samples were collected, including two species of plants (*S. glauca* and *S. salsa*), with four compartments, and each sample had six replicates. All soil and root samples were stored at -80°C until use.

### DNA extraction

All of the sample DNA was extracted by using the Wizard Genomic DNA Purification Kit (Promega, USA). The concentrations of genomic DNA were assessed using a NanoDrop 2000 spectrophotometer (Thermo Fisher Scientific, Waltham, USA) and qualities were evaluated by 0.8% agarose gel electrophoresis. High-quality DNA was stored at -80°C until amplicon sequencing.

For shotgun metagenomic sequencing, total DNA was directly extracted from 12 samples (including six bulk soils and six rhizoplane samples of two plants) using the standardized CTAB method (Wang et al., 2021b). DNA concentration and purity were examined by electrophoresis on 1% agarose gels. High-quality DNA was stored at -80°C until DNA sequencing.

### Amplicon sequencing and data processing

The hypervariable V5-V7 region of the 16S rRNA gene was amplified with the primers 779F (5’-AACMGGATTAGATACCCKG-3’)/1193R (5’-ACGTCATCCCCACCTTCC-3’) (Bodenhausen et al., 2013). The hypervariable ITS1-ITS2 region of the 18S rRNA gene was amplified with the primers ITS1F (5’-CTTGGTCATTTAGAGGAAGTAA-3’)/ITS2R (5’-GCTGCGTTCTTCATCGATGC-3’) (Adams et al., 2013). The PCRs (50 µL) were performed with 10 µL of 5 × FastPfu Buffer, 5 μL of 2.5 mM dNTPs, 1 μL of FastPfu polymerase, 2 µM primers, and ~10 ng of DNA. The PCR conditions were performed as previously described (Chelius and Triplett, 2001; Adams et al., 2013). Only samples with successful PCR amplifications were used for sequencing: 46 samples for bacteria except for *S. glauca*-BS4 and *S. glauca*-BS5 and 44 samples for fungi except for *S. salsa*-BS6, *S. glauca*-BS4, *S. glauca*-BS5, and *S. glauca*-BS6. Sequencing was performed on the Illumina MiSeq PE300 at Guangdong Magigene Biotechnology Co. Ltd. (Guangzhou, China).

The raw reads were processed by using the Quantitative Insights into Microbial Ecology II (QIIME II) pipeline (49). Raw data were assembled and quality-filtered by using DATA2, and assignment of taxonomy was performed by using SILVA (SSU138) for bacteria and UNITE (v8.3) databases for fungi (Koljalg et al., 2013; Quast et al., 2013). To reduce the influence of sequencing depths across samples, the sequences were rarefied based on the minimum numbers of samples for further analysis.

### Shotgun metagenomic sequencing and analysis

Sequencing libraries were generated using a NEBNext^®^ Ultra™ DNA Library Prep Kit for Illumina (NEB, California, USA). Metagenomic shotgun sequencing was performed on an Illumina NovaSeq 6000 platform at Shenzhen Weike Meng Technology Group Co., Ltd. (Shenzhen, China).

Raw data were quality filtered by removing adapters, and low-quality bases by using FastQC (v0.11.9) and Trimmomatic (v0.39). After filtering and removing host genes, clean reads were assembled by using Megahit with default parameters. All contigs longer than 300 bp were used for further analysis. Open reading frames (ORFs) in contigs were predicted by using Prodigal software (parameter: -p meta). Nonredundant genes were obtained using Cd-hit (Li and Godzik, 2006), and then the gene abundances were estimated in transcripts per million (TPM) using Salmon. To obtain the KEGG, GO, and COG annotation, the nonredundant protein sequence was then blasted to the EggNOG database by Eggnog-mapper software. To identify the species contained in the samples, Kraken2 was used, and the relative abundance of species in each sample was calculated by Bracken. The gene abundances involved in carbon, nitrogen, and sulfur cycling were identified by DiTing.

### Statistical analysis

The alpha diversity of the microbial community was calculated based on the normalized reads. The distinctions in alpha diversity between different samples were statistically compared using the t-test in GraphPad Prism (v8, San Diego, USA). Permutational multivariate analyses of variance (PERMANOVA) were performed to assess the influence of different factors on the microbial community based on the Bray-Curtis distance dissimilarity, and nonmetric multidimensional scaling (NMDS) analyses were used for data visualization. Moreover, differential enrichment analysis of microbial ASVs (relative abundance > 0.01%) was calculated by using “edgeR” in R. The ASVs with FDR-adjusted *P* < 0.05 and |log_2_ (fold changes) | >1 were considered as “enriched” or “depleted”, respectively. Venn diagrams of microbial ASVs across different niches were obtained using jvenn (Bardou et al., 2014). The variances in functional genes between bulk and rhizoplane soils were statistically compared using the t-test in GraphPad Prism version 8.

To assess the roles of determinism and stochasticity in the microbial community assembly process, the null model was used to calculate the beta nearest taxon index (βNTI) by the R “picante” library. A value of |βNTI| > 2 indicates a deterministic process (heterogeneous or homogeneous selection) (Stegen et al., 2013). In addition, |βNTI| < 2 indicates stochastic processes, including dispersal limitation [Bray-Curtis-based Raup-Crick matrix (RCbray) > 0.95], drift (|RCbray | < 0.95), and homogenizing dispersal (RCbray <-0.95) (Stegen et al., 2013).

Microbial network analysis was performed based on Spearman’s correlation coefficients using the Molecular Ecological Network Analysis Pipeline (http://ieg2.ou.edu/MENA) based on random matrix theory (Deng et al., 2012) and visualized by using Gephi (v0.9.2). To reduce the influence of rare ASVs, genera with relative abundances > 0.01% detected in at least one-third of the samples were included in the network analysis. The nodes with high degrees (top 20%) and low betweenness centrality (ranking in the bottom 20% of nodes with high degrees) were regarded as keystone genera in each network (Berry and Widder, 2014).

## Results

### Diversity of bacterial and fungal communities in two salt-tolerant plants

**Figure 1:**
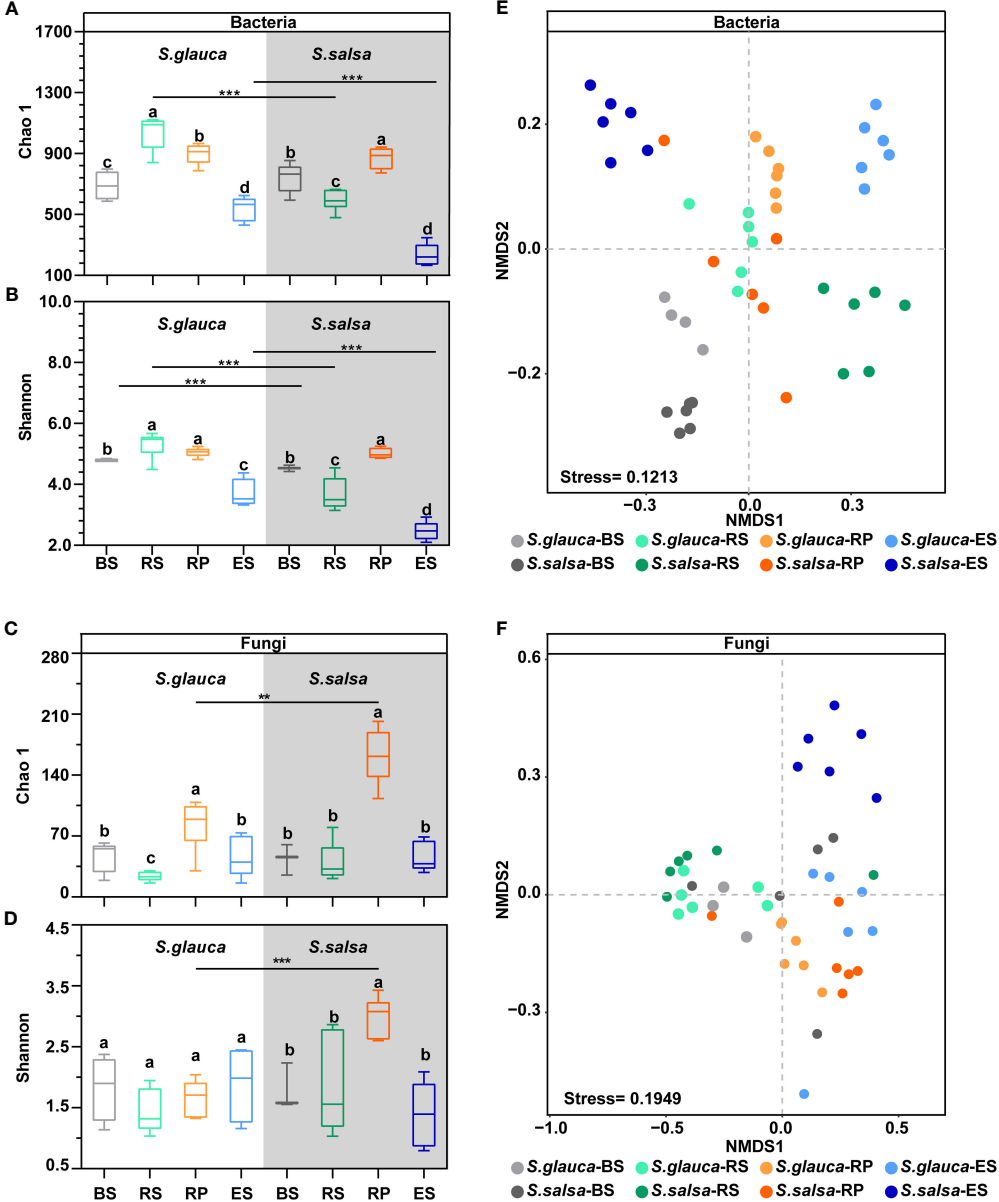
The alpha and beta diversity of bacterial and fungal communities in different samples. Chao 1 (**A** for bacteria, **C** for fungi) and Shannon (**B** for bacteria, **D** for fungi) indices of the microbial community in four compartments for two plants. BS, bulk soil; RS, rhizosphere; RP, rhizoplane; ES, endosphere. The asterisks (*) indicate significant variance between *S. glauca* and *S. salsa* within the same compartments. *P* value of < 0.05 (*), < 0.01 (**), or < 0.001 (***). Different lowercase letters over boxplots indicates statistically significant differences (t-test, P < 0.05) across niches within one plant. NMDS plot indicating the clustering of different samples for bacterial **(E)** and fungal **(F)** communities. Samples are color-coded based on four niches and two plants.

To identify the variances in microbial diversity and community composition between *S. glauca* and *S. salsa*, the 16S and ITS data from four root-associated compartments were analysed. After merging and filtering, a total of 42,676 and 34,594 sequences were obtained from the 16S and ITS raw reads, respectively, which were sorted into 22,403 ASVs for 16S and 1,220 ASVs for ITS data. The Good’s coverage values of bacterial and fungal data varied between 99.71% and 100.00% across all samples (**Table S1**), consistent with the species accumulation curve approaching an asymptote (**Figure S1**), suggesting that the sequencing effort recovered most of the diversity of local species. For the alpha diversity of the bacterial community, there were similar patterns between the two plants. Compared with the other three compartments, the alpha diversities of bacterial communities in the endospheres of *S. glauca* and *S. salsa* were the lowest (**Figures 1A, B**; P < 0.05, t-test). Additionally, the Chao 1 and Shannon indices in the rhizosphere of *S. glauca* were the highest among the four compartments and were significantly higher than those in the corresponding compartment of *S. salsa* (**Figures 1A, B**; *P* < 0.05, t-test). However, the fungi alpha diversity of the rhizoplane in *S. salsa* was significantly higher than that in *S. glauca* (**Figures 1C, D**). Moreover, for the fungal community, no notable variance in alpha diversity was found between *S. glauca* and *S. salsa*, except for the rhizoplane microbiomes (**Figures 1C, D**).

NMDS and PERMANOVA of the complete data set (i.e., 16S and ITS sequencing data for the four compartments of two plants) revealed that the difference in the microbial community was explained mainly by niches (R^2^ = 33.0%, *P <*0.05, bacteria; R^2^ = 15.5%, *P <*0.05, fungi) and then by the type of plant (R^2^ = 9.8%, *P <*0.05, bacteria; *R* ^2^ = 4.2%, *P <*0.05, fungi) (**Table 1**). NMDS revealed a clear and separate clustering of the samples from different niches (**Figures 1E, F**). We found marked differences in bacterial communities when the two plants were compared, with R^2^ values of 32.3% to 73.1%. Fungal communities were markedly different in the rhizoplane (R^2^ = 29.2%) and endosphere (R^2^ = 20.5%) between *S. salsa* and *S. glauca* (*P <*0.05). Furthermore, the niche explained significant variations in both bacterial and fungal communities (R^2^ value from 36.6% to 69.9%, *P <* 0.05) in the two plants (**Figures 1E, F**, **Table 1**).

**Table 1:** R^2^ and *P values* returned by PERMANOVA test for variance of microbial communities among different samples.

Factor(s)	Results by factor		Results by plant/niche		
	R^2^ (%)	*P value*	Comparison	R^2^ (%)	*P value*
Plant (Bacteria)	9.8	**0.001**	*S.salsa*-BS vs. *S.glauca-*BS	49.6	**0.004**
			*S.salsa*-RS vs. *S.glauca-*RS	47.8	**0.003**
			*S.salsa*-RP vs. *S.glauca-*RP	32.3	**0.003**
			*S.salsa*-ES vs. *S.glauca-*ES	73.1	**0.003**
Niche (Bacteria)	33.0	**0.001**	BS vs. RS vs. RP vs. ES (*S.glauca*)	59.3	**0.001**
			BS vs. RS vs. RP vs. ES (*S. salsa*)	69.9	**0.001**
Plant & niche (Bacteria)	68.7	**0.001**			
Plant (Fungi)	4.2	0.062	*S.salsa*-BS vs. *S.glauca-*BS	25.1	0.113
			*S.salsa*-RS vs. *S.glauca-*RS	4.2	0.803
			*S.salsa*-RP vs. *S.glauca-*RP	29.2	**0.003**
			*S.salsa*-ES vs. *S.glauca-*ES	20.5	**0.013**
Niche (Fungi)	15.5	**0.001**	BS vs. RS vs. RP vs. ES (*S.glauca*)	44.4	**0.001**
			BS vs. RS vs. RP vs. ES (*S. salsa*)	36.6	**0.001**
Plant & niche (Fungi)	42.5	**0.001**			

P value in bold indicate significant difference.

**Figure 2:**
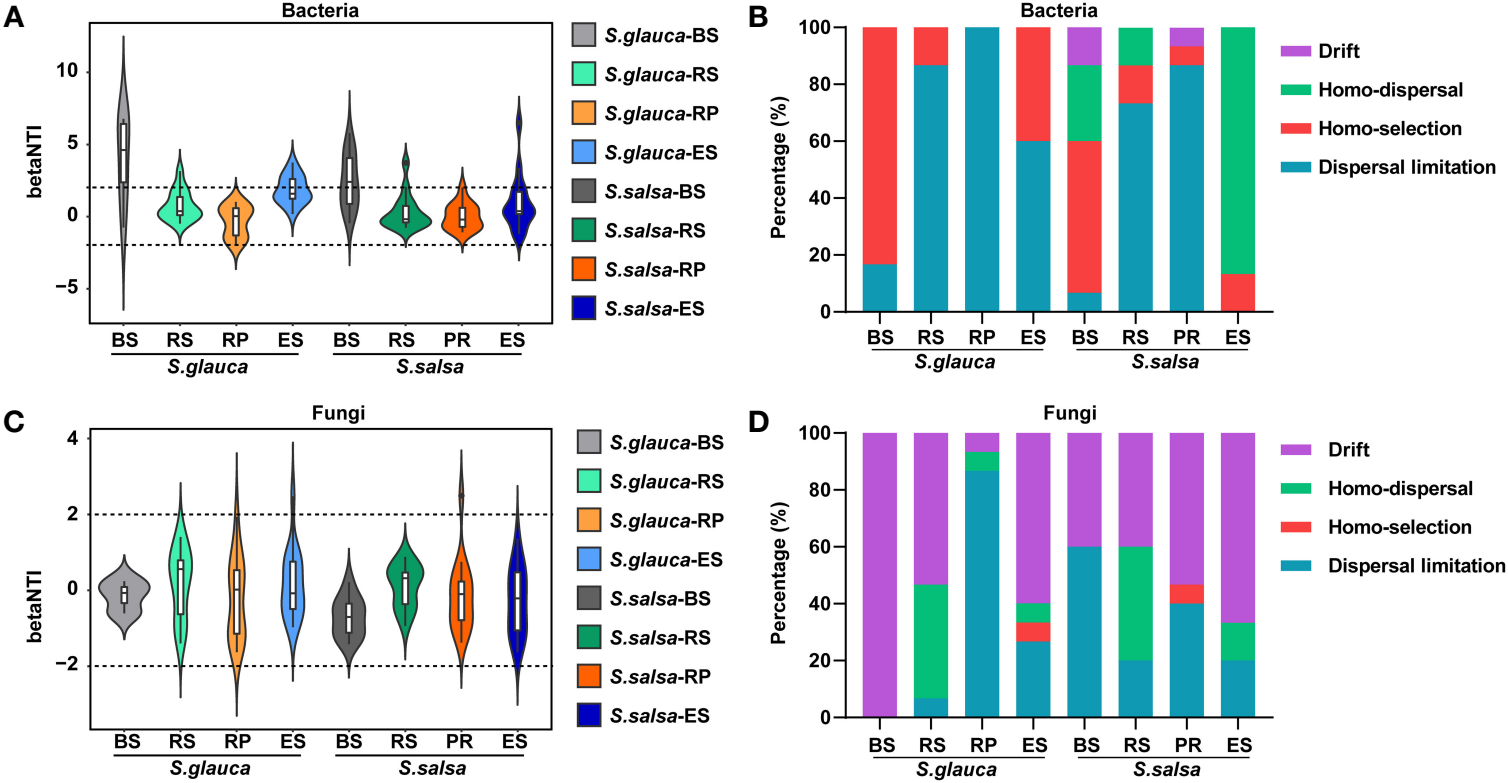
Deterministic or stochastic processes in bacterial **(A, B)** and fungal **(C, D)** communities of two plants among four compartments.

To explore the assembly mechanism of community composition, the null model was calculated. The results indicated that homo-selection belonging to deterministic process was the major process in bulk soil, accounting for 83.3% and 53.3% of the bacterial community variation across all samples in *S. glauca* and *S. salsa*, respectively. Although homo-dispersal is the main process in the endosphere of *S. salsa*, dispersal limitation is the main process for community assembly in other compartments of both plants. For the fungal community, drift was the important process in most compartments for the two plants, while dispersal limitation was the main process in the rhizoplane of *S. glauca* and bulk soil of *S. salsa*, accounting for 86.7% and 60.0% of fungal community variations across all samples. Collectively, the results showed that stochastic process contributed more effect on changes in microbial communities than deterministic process, and the contribution was slightly higher for *S. salsa* than for *S. glauca* (**Figure 2**).

### Variation in microbial community composition in different plants

**Figure 3:**
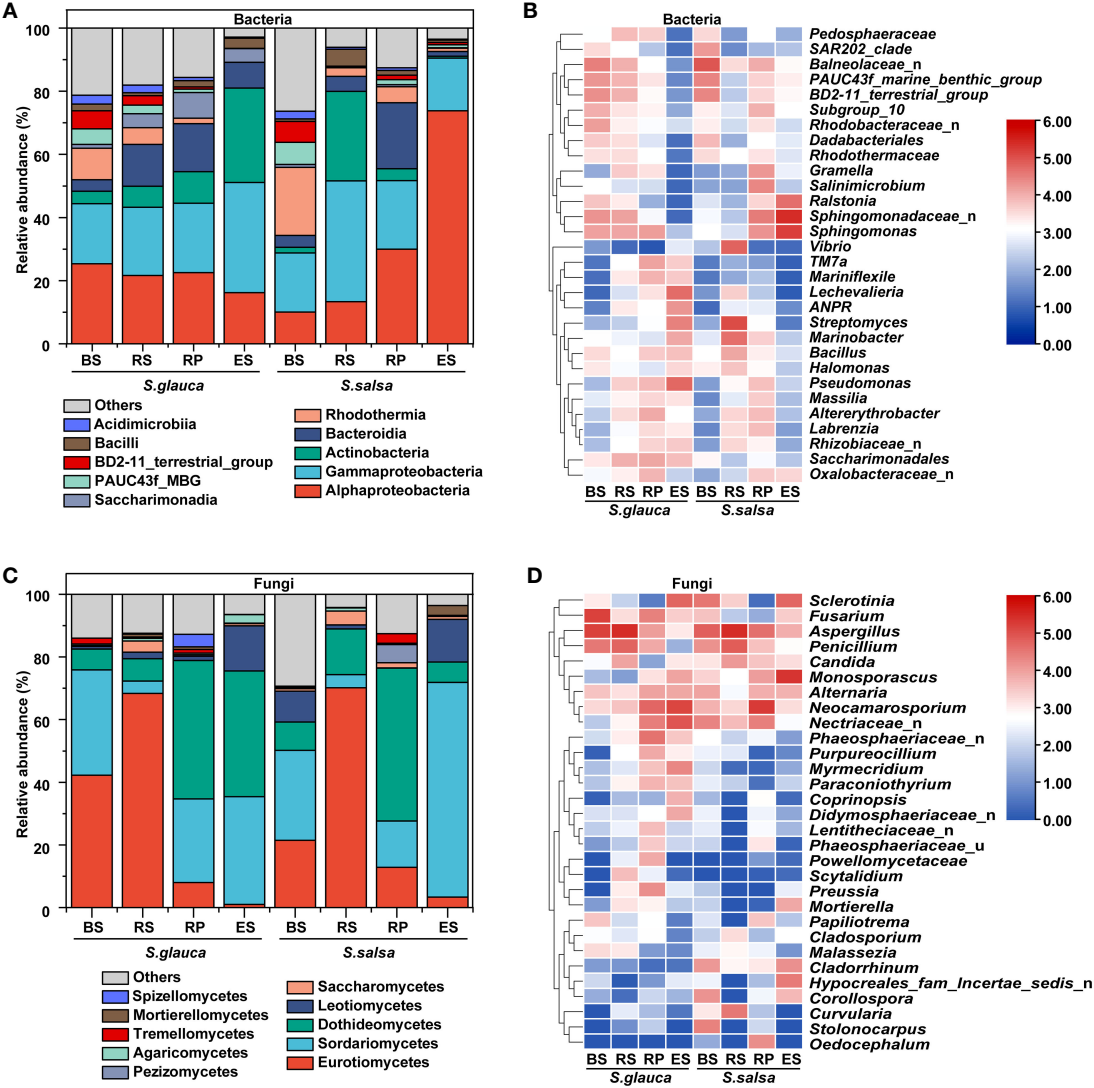
The relative abundances of dominant bacterial and fungal taxon at the class **(A, C)** and genus **(B, D)** levels.

In terms of community composition, there were marked variances in microbial classes and genera between the two plants. The dominant bacterial classes among the four compartments of different plants were Gammaproteobacteria, Alphaproteobacteria, and Actinobacteria (**Figure 3A**), with a dramatic variance in different samples. Alphaproteobacteria accounted for a relatively high proportion in other compartments of *S. glauca* (21.6%-25.3%), and the same situation appeared in the rhizoplane (30.0%) and endosphere (73.8%) of *S. salsa*. However, Actinobacteria occupied a high proportion in the endosphere of *S. glauca*, accounting for 29.9% of the whole community (**Figure 3A**). The fungal abundance at the class level had a similar distribution in the corresponding compartments of the two plants. Although there were differences among the different compartments, they were all concentrated in Eurotiomycetes, Sordariomycetes, and Dothideomycetes. The dominant classes of bulk soil were Eurotiomycetes and Sordariomycetes, the dominant class of the rhizosphere was Eurotiomycetes, and the dominant class of the rhizoplane was Dothideomycetes. Sordariomycetes was the dominant class shared by the endosphere of both plants, and Dothideomycetes was unique to *S. glauca* (**Figure 3C**).

**Figure 4:**
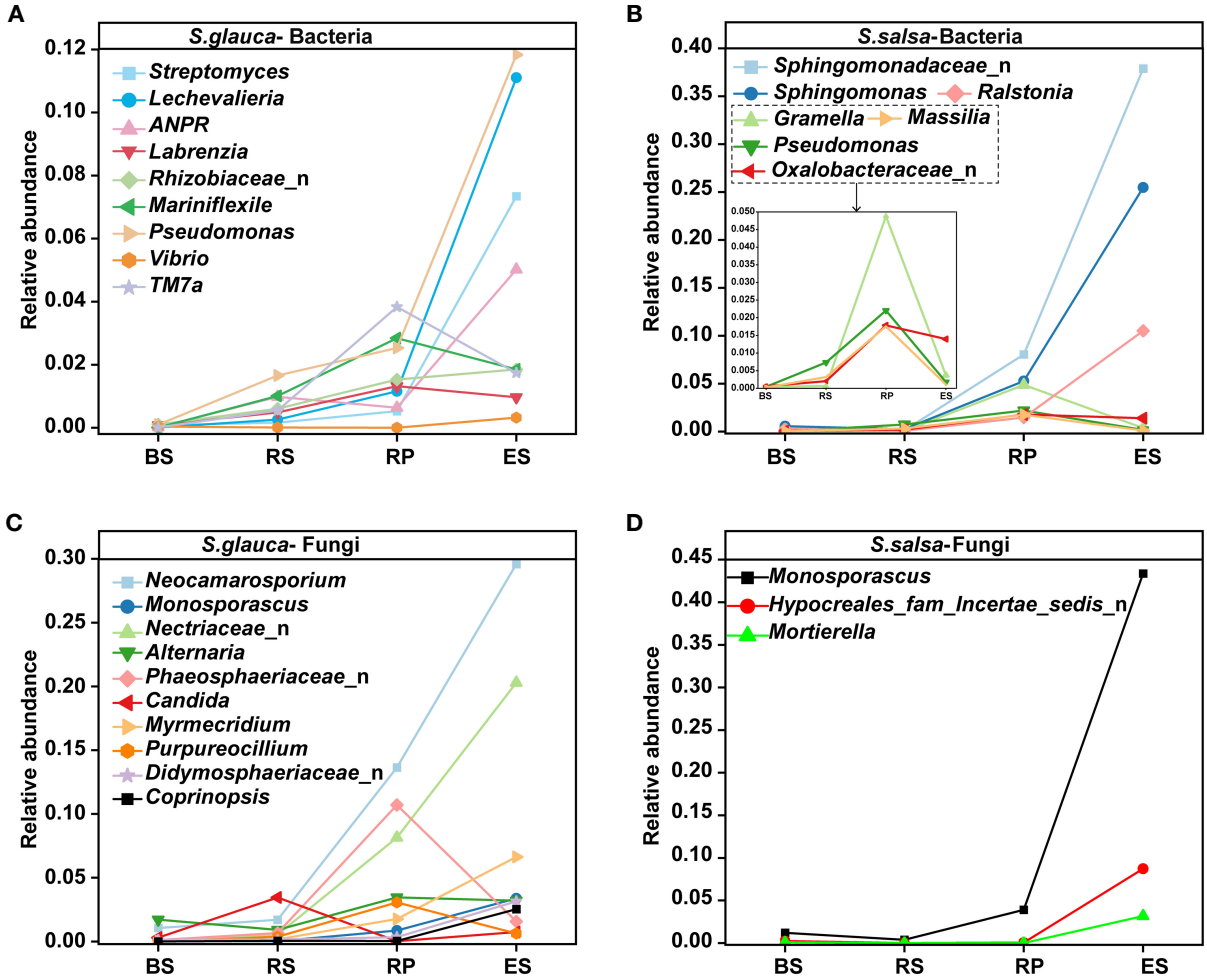
Changes in the relative abundances of dominant bacterial **(A, B)** and fungal **(C, D)** genera in different niches of *S. glauca* and *S. salsa*.

The variances in the microbial community composition were more obvious at genus level. *Sphingomonadaceae*_n, *Balneolaceae*_n, and PAUC43f_marine_benthic_group were the dominant groups in the bulk soils of *S. glauca*. The richness in the rhizosphere, rhizoplane, and endosphere decreased successively. *Streptomyces*, *Pseudomonas*, *Lechevalieria*, and *Rhizobiaceae*_n showed the opposite trend and were the most abundant in the endosphere. *Sphingomonadaceae*_n, *Sphingomonas*, and *Balneolaceae*_n dominated in the rhizosphere, while *Sphingomonas*, *Saccharimonadales*, and *Altererythrobacter* were the dominant groups in the rhizoplane. For *S. salsa*, *Sphingomonadaceae*_n and *Sphingomonas* were the dominant groups in the rhizoplane and endosphere. *Balneolaceae*_n, PAUC43f_marine_benthic_group, and BD2-11_terrestrial_group were the dominant groups in the bulk soils. *Streptomyces*, *Marinobacter*, and *Vibrio* dominated in the rhizosphere. Additionally, the relative abundances of *Pseudomonas*, *Allorhizobium-Neorhizobium-Pararhizobium-Rhizobium* (ANPR), *Rhizobiaceae*_n, and *Massilia* in other compartments of both plants were higher than those in their respective bulk soils (**Figure 3B**). Compared with bacteria, the community distribution of fungi at the genus level was more consistent, mainly focusing on *Aspergillus*, *Neocamarosporium*, and *Monosporascus*. Except for the endosphere, the dominant genera of fungi in the other compartments showed the same pattern. The dominant genus in bulk and rhizosphere soils was *Aspergillus*, and the dominant genus in the rhizoplane was *Neocamarosporium*. Moreover, the dominant genus in the endosphere of *S. glauca* was *Neocamarosporium*, while in the endosphere of *S. salsa* was *Monosporascus* (**Figure 3D**). Although the bacterial groups enriched by *S. glauca* and *S. salsa* in the endosphere were different, their classes were mainly concentrated in Alphaproteobacteria, Bacteroidia, and Gammaproteobacteria. Nine taxa, including *Lechevalieria*, ANPR, and *Mariniflexile*, were significantly enriched in the endosphere of *S. glauca*, while seven taxa, including *Sphingomonadaceae*_n and *Sphingomonas*, were significantly enriched in *S. salsa* (**Figures 4A, B**). The class of fungi enriched in the endosphere by the two types of plants mostly belonged to *Dothideomycetes* and *Sordariomycetes* (**Figures 4C, D**).

**Figure 5:**
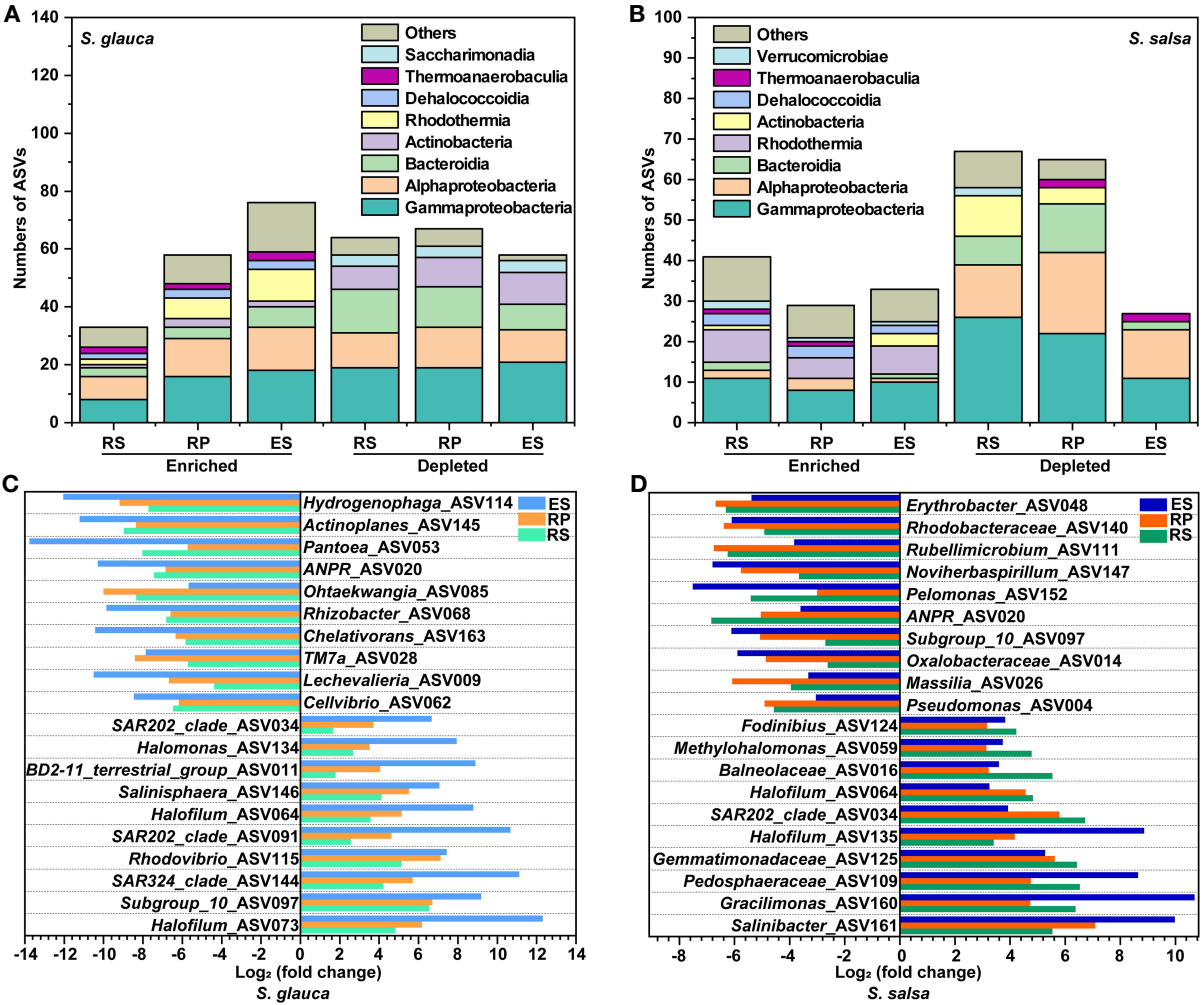
The enriched and depleted ASVs in bulk soils of *S. glauca* and *S. salsa*. Stacked bar charts indicate the taxonomy of enriched and depleted ASVs at the class level **(A, B)**. Shared enriched and depleted ASVs between microbes of bulk soils, rhizosphere, rhizoplane and endosphere at the genus level in *S. glauca* **(C)** and *S. salsa* **(D)**.

To further analyze the differences in microbial communities, enrichment and depletion due to bulk soil at the ASV level were calculated. In comparison, the bacteria in the endosphere of *S. glauca* were enriched for fewer ASVs while simultaneously depleting a larger proportion of ASVs (58 vs. 76) (**Figure S2**, **S3**). The rhizosphere of *S. salsa* was enriched and depleted for more ASVs (67 vs. 41) (**Figure S2**, **S3**). Among them, compared with other compartments, the bacterial ASVs enriched in bulk soils of the two plants mainly belonged to Gammaproteobacteria. In addition, many bacterial ASVs enriched in bulk soils of *S. glauca* belonged to Alphaproteobacteria, while those in *S. salsa* belonged to Rhodothermia. In the three compartments, the bacteria enriched by both plants were concentrated in Gammaproteobacteria, Alphaproteobacteria, and Bacteroidia (**Figures 5A, B**). In terms of enriched ASVs, 42 ASVs in *S. glauca* and 13 ASVs in *S. salsa* were shared by the three compartments (**Figure S3**). In *S. glauca*, the shared enriched ASVs of the top 10 were assigned to different genera, such as *Hydrogenophaga*, *Actinoplanes*, *Pantoea*, and ANPR (**Figure 5C**). Except for *Ohtaekwangia* and TM7a, which were more enriched in the rhizoplane, the other groups were more significantly enriched in the endosphere. In *S. salsa*, the shared ASVs were assigned to *Erythrobacter*, *Rhodobacteraceae*, *Rubellimicrobium*, *Noviherbaspirillum*, and so on (**Figure 5D**). For depleted ASVs, most communities were more significant in the endosphere (**Figures 5C, D**). Due to the low richness of fungal communities, differences in community between groups were not significant. In *S. glauca*, communities such as *Nigrospora* and *Purpureocillium_lilacinum* were enriched in the endosphere, while *Powellomycetaceae*, *Preussia_terricola*, and *Emerlopsis_minima* were enriched in the rhizoplane. Only *Aspergillus_templicola* was enriched in the endosphere in *S. salsa* (**Table S2**).

### Co-occurrence networks of two plants

**Figure 6:**
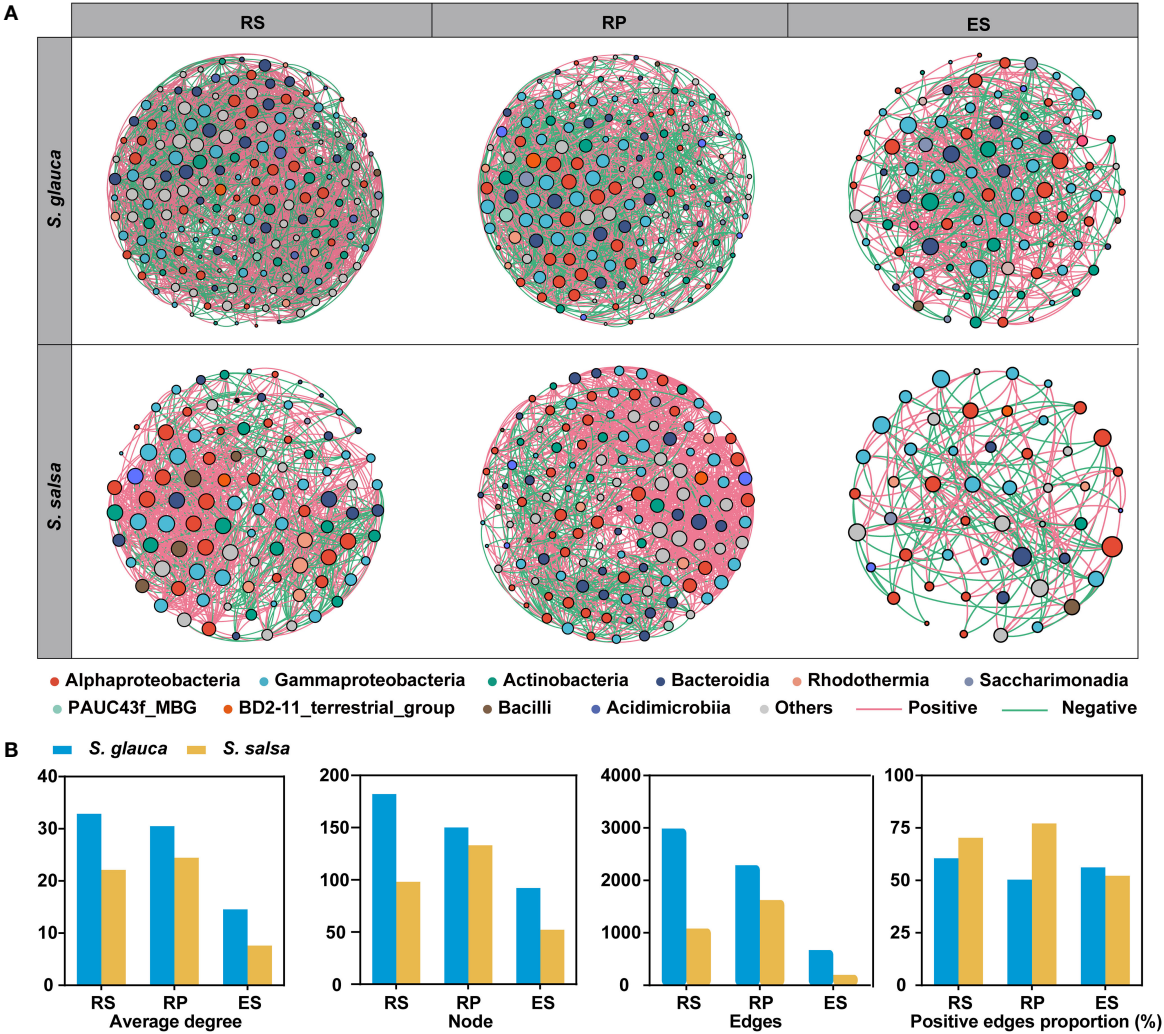
Distinct patterns of the bacterial co-occurrence networks between two plants in three niches. **(A)** Co-occurrence network of two plants in three niches. Nodes represent dominant bacterial genera that are shown in different colors. The node size is proportional to node degrees. **(B)** Topological parameters of network for three niche bacterial networks.

Co-occurrence network analysis was performed to compare the variances in microbial interactions between two plants across three root-related compartments. Consistent with bacterial diversity, network complexity was higher in the rhizosphere and rhizoplane than in the endosphere, with the highest average degree for networks in the rhizosphere of *S. glauca* (32.846) and the rhizoplane of *S. salsa* (24.451) and the lowest average degree in endosphere of *S. glauca* (14.565) and *S. salsa* (7.615) (**Figures 6A, B**, **Table S3**). The number of nodes and edges of the networks showed a clear downward trend from the rhizosphere to the endosphere, indicating a strong influence of niches on bacterial networks (**Figure 6B**, **Table S3**). Additionally, network complexity was higher in *S. glauca* than in *S. salsa*, with more nodes and edges and a higher average degree in the three compartments of *S. glauca* (**Figure 6B**, **Table S3**). Moreover, co-occurrence networks using dominant bacterial and fungal genera were also constructed, and the results showed that the patterns were similar to those of bacterial networks, with significantly reduced topological parameters in the endosphere, including nodes, edges, and average degree (**Figure S4**, **Table S4**). The results indicated that the networks of the rhizosphere and rhizoplane of the two plants were more robust and stable than those of the endosphere.

Based on node degree (top 20%) and betweenness centrality (bottom 20%), 38 keystone genera were identified, including 36 bacterial genera and two fungal genera (**Table S5**), which might participate in maintaining microbial community structure stability and plant tolerance. The majority of keystone genera belonged to bacterial classes, including Gammaproteobacteria (12), Alphaproteobacteria (9), and Bacteroidia (5). In addition, two fungal keystone genera (*Candida* and *Mortierella*) originated from the endosphere of *S. salsa* (**Table S5**). The keystone bacterial genera of the two plants varied in different compartments. Specifically, the keystone bacterial genera in the endosphere of *S. salsa* were *Marinobacter* and *Sphingomonas*, while those in *S. glauca* were *Defluviimonas* and *Methyloceanibacter* (**Table S5**).

### Specialized distinct functional profiles and potential mechanisms in alleviating salt stress

**Figure 7:**
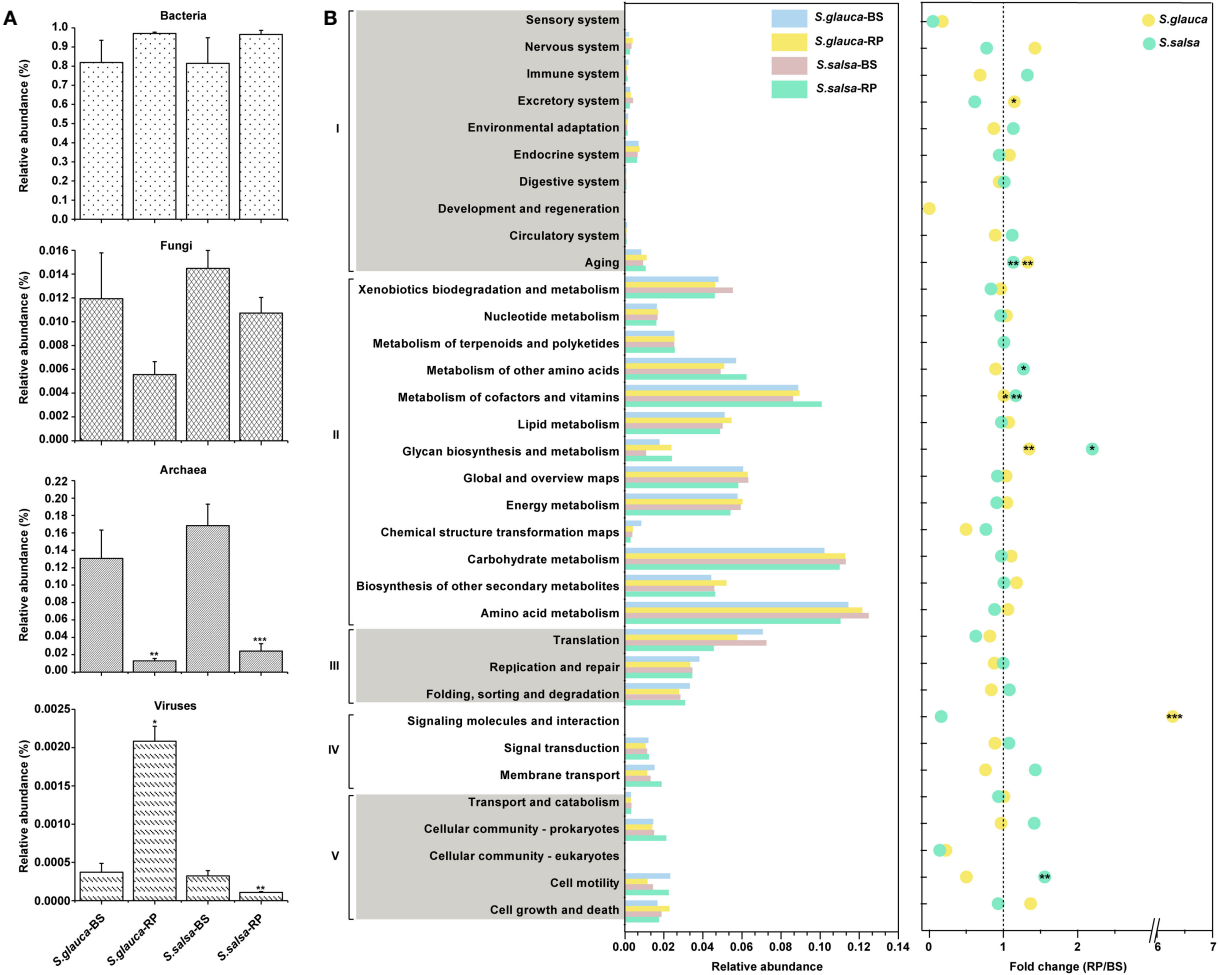
Taxonomic and functional profiles of microbiomes from shotgun sequencing. **(A)** The relative abundances of bacteria, fungi, archaea, and viruses in bulk and rhizoplane soils. **(B)** Relative abundances of genes annotated by KEGG databases and fold changes between bulk soils and rhizoplane soils. I, cellular processes; II, environmental information processing; III, genetic information processing; IV, metabolism; V, organismal systems. Stars in circles indicate pathways significantly enriched in rhizoplane soil. *P* value of < 0.05 (*), < 0.01 (**), or < 0.001 (***).BS, bulk soils; RP, rhizoplane.

To characterize the potential ecological roles of rhizoplane microbiomes under salt tolerance, 12 DNA samples from rhizoplane soils and bulk soils of two plants were used for metagenomic sequencing. The classification analysis of the metagenomic data showed that bacterial groups accounted for the majority of the soil microbial population, while archaea, viruses, and fungi accounted for a small proportion (<0.18 of total sequences) (**Figure 7A**). Specifically, compared to bulk soils, the relative abundance of fungi and archaea in the rhizoplane was lower in both plants. The virus in the rhizoplane of *S. glauca* was enriched, while the rhizoplane of *S. salsa* was depleted, compared with the relative abundance of virus in bulk soils (**Figure 7A**).

Based on the metagenomic data annotated in the KEGG database, we found that the relative abundance of categories of glycan biosynthesis and metabolism, metabolism of cofactors and vitamins, and aging in the rhizoplane were significantly higher than those in bulk soils (*P* < 0.05, t-test) (**Figure 7B**). Glycan synthesis is an essential functional trait, that is beneficial for carbohydrate metabolism and enhances plant stress resistance (**Figure 7B**). Thus, the relative abundances of genes related to glycan synthesis were compared between niches, and the results indicated that nearly all genes were more abundant in the rhizoplane than in bulk soils (**Figure S5**). Only genes involved in lipopolysaccharide and peptidoglycan biosynthesis were slightly lower in rhizoplane soils than in bulk soils of S. *glauca* (**Figure S5**).

**Figure 8:**
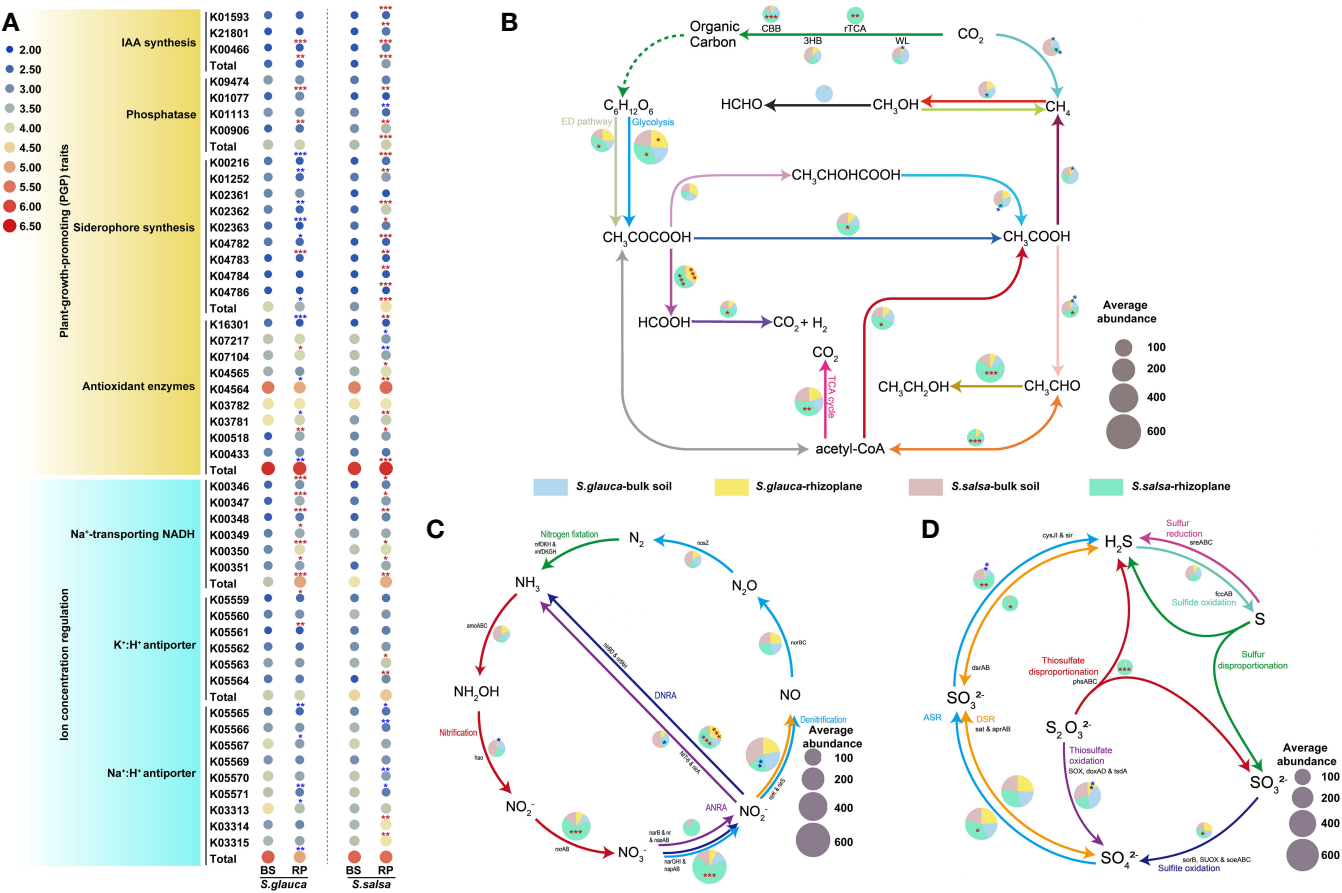
The variances in the relative abundances of genes involved in plant growth promotion and iron concentration regulation **(A)**, carbon **(B)**, nitrogen **(C)**, and sulfur **(D)** cycle among niches and plants. Red and blue stars indicated relative abundances of gene that were higher or lower in the rhizoplane niche than in bulk soils, respectively (*, P, 0.05; **, P, 0.01). The pie chart size **(B, C, D**) is proportional to gene abundance related to the pathway. BS, bulk soil; RP, rhizoplane. DNRA, dissimilatory nitrate reduction to ammonia; ANRA, assimilatory nitrate reduction to ammonia.

To further explore the potential function of the rhizoplane microbiomes in mitigating the impacts of salt on plant growth, genes related to plant growth promotion and ion concentration regulation were investigated. The results indicated that microbiomes in the rhizoplane of *S. salsa* had a higher proportion of plant growth-promoting (PGP) trait-related genes, including IAA biosynthesis, siderophore synthesis, phosphatase, and antioxidant enzymes compared with bulk soils (**Figure 8A**). However, microbiota in the rhizoplane of *S. glauca* was enriched in genes related to IAA biosynthesis and phosphatase, while genes related to siderophore synthesis and antioxidant enzymes were significantly lacking (**Figure 8A**). The results suggested that the microbial communities in the rhizoplane of the two plants may adopt different ways to regulate plant tolerance, thereby affecting plant growth. Moreover, genes related to potassium and sodium ions transport were reported to directly affect salt toxins in plants. The relative abundances of K^+^/H^+^ and Na^+^ transport-related genes in the rhizoplane of *S. glauca* and *S. salsa* were higher than those in bulk soils, while Na^+^/H^+^ antiporter genes exhibited opposite trends, indicating that changes in the genes involved in concentration regulation may enhance plant tolerance by reducing plant salt toxins (**Figure 8A**).

Microbial metabolism can provide nutrients for soil, promote the material cycle of carbon, nitrogen, sulfur, and other elements, and promote the healthy growth of plants. Functional genes related to carbon, nitrogen, and sulfur cycling indicated markedly different distribution patterns among niches and different plants (**Figure 8B**). Compared with those in bulk soils, the genes related to carbon fixation were more abundant in the rhizoplane compartment of *S. salsa*. Additionally, the enrichment of process genes such as glycolysis showed a stronger metabolic process in both plants (**Figure 8B**). The genes responsible for the dissimilar nitrate reduction to ammonium (DNRA) pathway were more abundant in rhizoplane soils than in bulk soils, producing plant recyclable ammonium salts by dissimilating and reducing nitrate nitrogen (NO_3_^-^). Compared to bulk soils, most processes involved in nitrogen cycling (e.g., *narG*, *napA*) were more abundant in the rhizoplane, especially in *S. salsa* (**Figure 8C**). For the genes involved in the sulfur cycle, the genes involved in the process of sulfite oxidation and assimilatory sulfate reduction to sulfide were enriched in the rhizoplane of *S. salsa* compared with bulk soils (**Figure 8D**).

## Discussion

As pioneer plants in saline-alkali land, *S. glauca* and *S. salsa* play a key role in soil bioremediation (Cui et al., 2023; Wang et al., 2023), but the distribution of plant-related microbial communities and functional genes alleviating high salt stress are still unclear. This study provided in-depth research on the characteristics and functional genes of salt-tolerant microbiomes. The results indicated that the root-associated niches of the two plants have different dominant microbial community distributions and keystone species. Metagenomic analysis showed gene enrichment related to PGP traits (such as phosphate dissolution) and Na^+^ concentration regulation in the two plants, but the distribution of genes involved in carbon, nitrogen, and sulfur cycling significantly differed. The results indicated that the two plants might have adopted different salt tolerance mechanisms, greatly enhancing the understanding of the root-related microbial assembly mechanisms of *S. glauca* and *S. salsa* and their ecological roles in plant adaptation to saline-alkali soils.

### Plant-specific community assembly pattern in root-related compartments

Understanding the assembly mechanism of the microbial community is important for the study of the microbial ecological roles of salt-tolerant plants. Our results showed that the assembly of microbial communities was mainly driven by stochastic process (**Figure 2**), and greater roles of stochasticity suggested smaller environmental gradients or weak impacts of environmental variances (Liu et al., 2020b). In the bulk soils of these two plants, the bacterial communities were dominated by homo-selection, while the root soil contributed more by stochastic processes (mainly dispersal limitation and homo-dispersal) (**Figure 2**), which might indicate that bacterial communities close to roots had a higher ability to adapt to changes in soil conditions (Cheng et al., 2023). The fungal communities were also dominated by stochastic processes of drift, homo-dispersal, and dispersal limitation. Moreover, compared with *S. glauca*, a stronger stochasticity in the microbial community assembly was found in *S. salsa* (**Figure 2**), indicating that the microbial community of *S. salsa* was less sensitive to variations in soil parameters and thus more adaptable to saline soils (Yang et al., 2021; Xu et al., 2022).

Network analysis has been applied to identify keystone taxonomic groups or functions within microbial communities (Michel-Mata et al., 2022). We built a co-occurrence network of root-associated niches in different plants (**Figure 6**). The more nodes and edges in the networks of the rhizosphere and rhizoplane indicated more complex and robust microbial co-occurrences than those in the endosphere (Cheng et al., 2023). The genomes of rhizosphere and endosphere bacteria associated with *S. salsa* are enriched in genes involved in salt stress acclimatization, nutrient solubilization and competitive root colonization (Yuan et al., 2016). The keystone species of bacteria were mainly assigned to Gammaproteobacteria and Alphaproteobacteria (**Table S5**), consistent with the results of community composition and community differences in ASV levels (**Figures 3A**, **5**). As generally found, Gammaproteobacteria and Alphaproteobacteria communities in the endosphere tended to be phylogenetically assembled (Gupta et al., 2022), indicating that the aggregation of these groups near the roots might play important roles in plant saline-alkali tolerance.

Faced with complex environments, plants might recruit specific microorganisms to alleviate stress. Research has shown that beneficial microorganisms attracted by plants serve as key members of the plant microbiome to enhance their resistance to adverse environments (Zheng et al., 2021). *Pseudomonas* and *Sphingomonas* have been reported to promote plant growth and/or prevent abiotic stresses (Guo et al., 2021; Pallavi et al., 2023). *Pseudomonas* and *Sphingomonas* of these two plants were the dominant groups in the root-associated soils in this study and were enriched in the endosphere (**Figures 3B**, **4A, B**), suggesting a core role of them in resisting salt stress. Beneficial fungal species can alter the fungal community composition and promote the abundance and interactions of fungi in soil (Nanjundappa et al., 2019). For example, the fungus *Neocatarosporium* can enhance plant tolerance to salt-alkali by increasing plant proline content and antioxidant enzyme activity (Hosseyni Moghaddam et al., 2021; Hosseyni Moghaddam et al., 2022). Although fungi had low abundance, *Neocatarosporium* occupied a dominant position and was enriched in the endosphere of *S. glauca* in this study (**Figures 3D**, **4C**), suggesting that this endophytic group might participate in improving plant resistance ability to salt stress. Moreover, *Monosporascus* has been reported to maximize the accumulation of soil organic matter and cause soil acidification, which helps improve plant growth performance and ecological adaptability (Zuo et al., 2022). In *S. salsa*, *Monosporascus*, as a dominant genus, was significantly enriched in the endosphere (**Figures 3D**, **4D**), suggesting that this genus might play important roles in the plant carbon cycle, nutrient absorption, and neutralizing the alkalinity of saline-alkali soil.

### Distinct functional profiles of the rhizoplane microbiomes in two plants

Soil salinization not only inhibits the grassing process but the accumulation of soil organic matter, resulting in a loss of nutrient contents, including nitrogen, phosphorus, and organic matter, thereby affecting the physicochemical characteristics of the soil (Padhy et al., 2022). The biogeochemical processes of carbon, nitrogen, and sulfur play a unique role in the material cycle and energy flow of coastal ecosystems, in which the driving role of microorganisms is widely considered (Liao et al., 2021; Palit et al., 2022). Our results showed that the fixation pathways of CO_2_ mainly included the Calvin cycle (CBB cycle) and the reduced citric acid cycle (rTCA cycle) (**Figure 8B**). The rhizoplane of *S. salsa* has a richer carbon fixation gene composition, which might increase the content of available carbon sources. Additionally, while the accumulation of soluble sugar is conducive to the removal of reactive oxygen species (Orozco-Mosqueda et al., 2019), in order to protect key organelles in plants and might play important roles in the metabolism of carbon compounds and salt tolerance of plants (Guo et al., 2021).

Multiple bacterial groups related to nitrogen fixation were found, including *Rhizobiaceae* (e.g., *Allorhombium*-*Neorhombium Pararhombium*-*Rhizobium*), *Oxalobacteraceae* (e.g., *Massilia*) and *Pseudomonas*, and the relative abundances were higher in the rhizoplane than in bulk soils (**Figures 3B**, **4A, B**), which may be conducive to the transformation and utilization of nitrogen. For example, as notable nitrogen fixers, *Rhizobium* bacteria are closely related to the nitrogen cycle process, including nitrogen fixation, denitrification and nitrification (Balázs et al., 2021; Padda et al., 2022). *Rhizobium* sp. ARR11 enhances root nitrogen absorption and promotes root growth by producing IAA and nitrogenase activity (Chai et al., 2022). *Pseudomonas stutzeri* A1501 changed the community composition of diazotrophs in the rhizosphere, improving the growth of corn (Ke et al., 2019). Enriched *Oxalobactereae* can improve the growth performance of maize under nitrogen deficiency conditions (Yu et al., 2021). Additionally, the genes involved in the DNRA pathway were significantly enriched in the rhizoplanes of both plants (**Figure 8C**), which is beneficial for the accumulation of nitrogen in the rhizoplane. The DNRA process is the main pathway of nitrogen transformation and one of the potentially important nitrogen cycle processes in soil ecosystems (Pandey et al., 2020). The nitrogen cycle process of rhizoplane microorganisms of *S. salsa* showed a similar trend with the carbon fixation pathway, which was markedly higher than that of *S. glauca* (**Figure 8C**).

For sulfur cycling, no significant difference in the production of sulfate between different compartments was found, but microorganisms in the rhizoplane of *S. salsa* generated more H_2_S (**Figure 8D**). Although H_2_S is considered a toxic gas, an increasing number of studies have proven that H_2_S-mediated thiometabolism plays a unique role in plant growth and development, especially in biotic and abiotic stress responses (Liu et al., 2021). H_2_S has been found to enhance antioxidant enzyme activity (Kaya et al., 2018) and increase the content of endogenous NO and total S-nitrosothiols (SNOs) in plants under high salt stress, as well as the activities of nitrate reductase and glyoxylase I and II (Ziogas et al., 2015) (Janicka et al., 2018), thus improving the resistance of plants to high salinity/alkalinity. *S. glauca* and *S. salsa*, as pioneer plants for vegetation restoration in saline-alkali land, showed different physiological differences in their adaptability. *S. glauca* seedlings have a higher alkali tolerance than *S. salsa*, while *S. salsa* has a higher salt tolerance (He et al., 2012). The difference in stress resistance between the two plants is mainly due to the adaptability of the root system and the different selective absorption abilities of the root system to inorganic ions (Li et al., 2019). The rhizoplane of *S. salsa* has more abundant functional genes involved in carbon, nitrogen, and sulfur cycles, suggesting that its microbial community has a stronger metabolic process, indicating that *S. salsa* may have a stronger environmental adaptability.

Many root-associated microorganisms, especially PGPR, have genes related to phosphatase, IAA biosynthesis, and antioxidant enzymes, which can improve the salt resistance ability of plants (Singh et al., 2017; Kaya et al., 2020). For instance, phosphatase was proven to convert nutrients into small molecules, thereby improving the absorption of nutrients by roots (Daloso et al., 2017). The genes encoding phosphatases were more abundant in the rhizoplanes of the two plants than in bulk soils in this study. Under salt stress, excessive production of reactive oxygen species can lead to lipid peroxidation (Yuan et al., 2018). The main common free radical produced in plants is O_2_·^−^.As a primary defensive enzyme, SOD could catalyse the conversion of O_2_·^−^ to H_2_O_2_, and CAT and POD could degrade H_2_O_2_ (Panda et al., 2019). *S. salsa* has a higher level of antioxidant enzyme system-related genes in its rhizoplane microbiota, with more abundant genes encoding SOD (e.g., K04565 and K04564) and CAT (e.g., K03781) (**Figure 8A**). Salt stress can lead to disorders of micronutrients, such as iron deficiency (Ashraf et al., 2023), and the enrichment of genes related to iron carrier production in the rhizoplane microbiota of *S. salsa* could alleviate this symptom. In our work, genes encoding proteins related to the above processes (at least one plant) were more abundant in rhizoplanes than in bulk soils, similar to previous findings (Chen et al., 2019; Shao et al., 2019; Zheng et al., 2021). Moreover, *S. salsa* has a richer distribution of genes involved in PGP traits than *S. glauca*, suggesting that *S. salsa* might be more conducive to enriching microbes with the potential to improve environmental adaptability and promote its growth (**Figure 8A**). This is similar to the results of carbon, nitrogen, and sulfur cycling, suggesting that *S. salsa* may be more adaptable to the extreme environment of the saline-alkali soils.

Excessive Na^+^ was found to trigger overproduction of ROS and damage plant organelles (Ma et al., 2023). Betacyanin is a secondary metabolite produced by plants under stress (Li et al., 2023), and its production and changes have a stronger correspondence and correlation with the environment than primary metabolites. Research has shown that betacyanin provides photoprotective light screening and potentially functions as a ROS-scavenger (Gengatharan et al., 2015; Zhou et al., 2021). Compared to *S. glauca*, *S. salsa* could accumulate more betacyanin to resist harsh environments with high salt content. Additionally, the inferior salt resistance of *S. glauca* compared to *S. salsa* might result from the poorer root K maintenance ability under high salinity (Li et al., 2019). Thus, *S. salsa* was more tolerant to salt stress than *S. glauca*. In this process, Na^+^ transport plays an important role in plant survival under high salinity conditions (van Zelm et al., 2020). A high salinity tolerance of plants is related to lower concentration of Na^+^ accumulated in branches or leaves (Chen and Polle, 2010). *S. glauca* and *S. salsa* both exhibited a low abundance of Na^+^/H^+^ antiporter genes and a high abundance of Na^+^ transporter genes in their rhizoplane microbes, which allows them to retain Na^+^ in cells and reduce the Na^+^ content in plants (**Figure 8A**). The salt tolerance of microorganisms is significantly higher than that of plant cells, and some microorganisms can even tolerate 4 M NaCl (Kearl et al., 2019). Therefore, root-related microorganisms can protect the host from high Na^+^ concentrations, which is consistent with previous studies showing that poplar salt tolerance is closely related to reduced Na^+^ uptake. Furthermore, maintaining K^+^/Na^+^ homeostasis is also important for plants to resist salt stress (Ma et al., 2023). Maintaining K^+^/Na^+^ ratios is a key pathway for tolerating salt-induced osmotic stress when Na^+^ enters plant cells (van Zelm et al., 2020). The rhizoplane microbes of both plants exhibit a high abundance of K^+^/H^+^ antiporter genes, which may increase the content of K^+^ and maintain the K^+^/Na^+^ ratios in plant cells (**Figure 8A**), thereby reducing the disruption of enzymatic activities and inhibition of related protein synthesis in a high salt environment (Almeida et al., 2017). Moreover, the relative abundance of Na^+^ transporter genes was higher of rhizoplane microbes in *S. glauca* than in *S. salsa*, which might have played a compensatory role in the survival of *S. glauca* in saline-alkali soil to enhance its tolerance to the environment. Notably, the conclusions were obtained using metagenomic sequencing and therefore should be interpreted with caution because they could not guarantee the expression of key genes. To gain mechanistic insight into the microbial response to saline stress, targeted methods, such as implementing metatranscriptomics and RT-qPCR, will be needed to verify the expression of key genes and pathways in saline soil.

## Conclusion

Our work investigated the microbial community assembly and functional profiles of root-related microbiomes of two salt-tolerant plants and revealed a distinct microbial community compositions and functional gene distributions in different compartments. The keystone species belonging to *Pseudomonas* and *Sphingomonas* played significant roles in sustaining plants survival under salt stress. In addition, microbiomes in the rhizoplane soils have more abundant genes related to plant growth promotion and salt stress defense than those in bulk soils, especially in salt-tolerant *S. salsa*. Moreover, the genes involved in carbon fixation, DNRA, and sulfite oxidation were enriched in the rhizoplane of *S. salsa*, which might participate in defending against salt stress and promoting plant growth. Our findings highlight the community assembly and functional variances of microbiomes in root-associated niches of salt-tolerant plants in saline-alkali environments and lay foundations for the potential use of keystone species to promote plant growth and develop economic value in saline-alkali soils.

## Data availability statement

The datasets presented in this study can be found in online repositories. The names of the repository/repositories and accession number(s) can be found below: https://www.ncbi.nlm.nih.gov/genbank/, PRJNA994656; https://www.ncbi.nlm.nih.gov/genbank/, PRJNA994665; https://www.ncbi.nlm.nih.gov/genbank/, PRJNA994852.

## Author contributions

LT: Conceptualization, Funding acquisition, Methodology, Writing – original draft, Writing – review & editing. LZ: Data curation, Investigation, Writing – review & editing. YH: Investigation, Visualization, Writing – review & editing. ZW: Writing – review & editing, Investigation. LD: Data curation, Writing – review & editing. ZZ: Supervision, Writing – review & editing.
