# Supplementary material for: Microbial community assembly and functional profiles along the soil-root continuum of salt-tolerant Suaeda glauca and Suaeda salsa
Source: Front Plant Sci. 2023 Nov 17;14:1301117. doi: 10.3389/fpls.2023.1301117 (PMC10691491; doi:10.3389/fpls.2023.1301117)
Supplement: Supplementary file 1 [file DataSheet_1.docx]

Supplementary material for

### Microbial community assembly and functional profiles along the soil-root continuum of salt-tolerant *Suaeda glauca* and *Suaeda salsa*

**Table S1** The diversity (Shannon and Simpson) indices, richness (Chao Ⅰ and ACE) indices and Good’s coverage across all samples

| Sample | Bacteria | | | | | Fungi | | | | |
| --- | --- | --- | --- | --- | --- | --- | --- | --- | --- | --- |
|  | Shannon | Simpson | Chao1 | ACE | Good’s_coverage | Shannon | Simpson | Chao1 | ACE | Good’s_coverage |
| *S. glauca*-BS1 | 4.74 | 0.97 | 643.31 | 623.07 | 0.9986 | 1.58 | 0.71 | 25.00 | 25.00 | 1.0000 |
| *S. glauca*-BS2 | 4.73 | 0.98 | 797.86 | 798.88 | 0.9983 | 1.56 | 0.67 | 45.75 | 50.94 | 0.9998 |
| *S. glauca*-BS3 | 4.83 | 0.98 | 731.55 | 727.06 | 0.9986 | 2.24 | 0.82 | 60.00 | 59.76 | 0.9999 |
| *S. glauca*-BS4 | 4.85 | 0.98 | 587.27 | 555.40 | 0.9993 |  |  |  |  |  |
| *S. glauca*-RS1 | 5.67 | 0.99 | 1122.61 | 1117.85 | 0.9971 | 1.03 | 0.50 | 28.00 | 26.44 | 0.9999 |
| *S. glauca*-RS2 | 5.44 | 0.99 | 1036.30 | 1031.88 | 0.9976 | 1.56 | 0.64 | 34.00 | 37.43 | 0.9998 |
| *S. glauca*-RS3 | 5.22 | 0.99 | 840.92 | 846.12 | 0.9983 | 2.71 | 0.87 | 80.00 | 80.21 | 1.0000 |
| *S. glauca*-RS4 | 5.52 | 0.99 | 1090.43 | 1082.09 | 0.9973 | 2.86 | 0.86 | 148.67 | 148.48 | 0.9999 |
| *S. glauca*-RS5 | 5.51 | 0.99 | 1111.16 | 1099.39 | 0.9972 | 0.74 | 0.42 | 21.00 | 26.54 | 0.9998 |
| *S. glauca*-RS6 | 4.49 | 0.94 | 575.44 | 568.48 | 0.9995 | 1.34 | 0.65 | 32.00 | 28.67 | 0.9999 |
| *S. glauca*-RP1 | 4.97 | 0.98 | 858.48 | 847.95 | 0.9980 | 3.16 | 0.91 | 201.67 | 201.97 | 0.9997 |
| *S. glauca*-RP2 | 5.13 | 0.98 | 930.06 | 929.47 | 0.9976 | 3.16 | 0.91 | 185.46 | 185.99 | 0.9999 |
| *S. glauca*-RP3 | 5.02 | 0.99 | 787.82 | 798.38 | 0.9981 | 2.63 | 0.87 | 113.00 | 113.22 | 1.0000 |
| *S. glauca*-RP4 | 5.12 | 0.98 | 895.16 | 896.55 | 0.9977 | 2.60 | 0.84 | 146.09 | 145.61 | 0.9996 |
| *S. glauca*-RP5 | 4.82 | 0.97 | 948.11 | 945.92 | 0.9974 | 3.43 | 0.94 | 172.00 | 172.12 | 0.9999 |
| *S. glauca*-RP6 | 5.25 | 0.99 | 965.35 | 960.37 | 0.9975 | 2.99 | 0.89 | 151.17 | 146.63 | 0.9997 |
| *S. glauca*-ES1 | 3.46 | 0.90 | 561.63 | 557.96 | 0.9977 | 1.83 | 0.78 | 39.20 | 40.30 | 0.9999 |
| *S. glauca*-ES2 | 3.37 | 0.90 | 463.41 | 465.05 | 0.9986 | 0.89 | 0.35 | 28.00 | 29.56 | 0.9999 |
| *S. glauca*-ES3 | 4.38 | 0.97 | 624.69 | 610.09 | 0.9987 | 1.17 | 0.51 | 63.11 | 65.02 | 0.9997 |
| *S. glauca*-ES4 | 4.12 | 0.95 | 570.14 | 562.57 | 0.9988 | 0.80 | 0.36 | 34.00 | 34.97 | 0.9999 |
| *S. glauca*-ES5 | 3.59 | 0.93 | 596.30 | 588.84 | 0.9978 | 2.09 | 0.81 | 37.00 | 38.30 | 0.9999 |
| *S. glauca*-ES6 | 3.32 | 0.91 | 429.69 | 439.86 | 0.9987 | 1.62 | 0.65 | 69.00 | 69.00 | 1.0000 |
| *S. salsa*-BS1 | 4.52 | 0.97 | 801.52 | 777.57 | 0.9971 | 1.14 | 0.53 | 19.00 | NA | 0.9999 |
| *S. salsa*-BS2 | 4.55 | 0.97 | 853.43 | 845.45 | 0.9972 | 2.38 | 0.83 | 62.00 | 62.00 | 1.0000 |
| *S. salsa*-BS3 | 4.42 | 0.97 | 593.51 | 598.57 | 0.9994 | 2.06 | 0.78 | 38.00 | 39.00 | 1.0000 |
| *S. salsa*-BS4 | 4.52 | 0.97 | 672.63 | 681.71 | 0.9980 | 0.83 | 0.32 | 55.50 | 55.49 | 0.9999 |
| *S. salsa*-BS5 | 4.52 | 0.97 | 762.27 | 765.44 | 0.9977 | 1.74 | 0.69 | 56.00 | 56.00 | 1.0000 |
| *S. salsa*-BS6 | 4.63 | 0.97 | 767.00 | 767.85 | 0.9981 |  |  |  |  |  |
| *S. salsa*-RS1 | 3.39 | 0.88 | 599.00 | 587.22 | 0.9979 | 1.32 | 0.59 | 26.00 | 24.53 | 0.9999 |
| *S. salsa*-RS2 | 2.73 | 0.77 | 478.82 | 458.30 | 0.9986 | 1.03 | 0.54 | 20.33 | 20.98 | 0.9999 |
| *S. salsa*-RS3 | 4.54 | 0.96 | 664.03 | 659.51 | 0.9992 | 1.94 | 0.81 | 28.50 | 29.95 | 0.9999 |
| *S. salsa*-RS4 | 3.15 | 0.82 | 661.08 | 657.21 | 0.9981 | 1.68 | 0.64 | 30.00 | 30.64 | 1.0000 |
| *S. salsa*-RS5 | 3.86 | 0.94 | 582.33 | 589.79 | 0.9985 | 0.81 | 0.43 | 16.00 | 18.75 | 0.9999 |
| *S. salsa*-RS6 | 3.49 | 0.86 | 571.52 | 572.25 | 0.9991 | 1.28 | 0.60 | 20.50 | 21.23 | 0.9999 |
| *S. salsa*-RP1 | 5.04 | 0.98 | 941.58 | 935.80 | 0.9977 | 1.34 | 0.61 | 84.75 | 86.87 | 0.9997 |
| *S. salsa*-RP2 | 3.96 | 0.94 | 772.82 | 768.78 | 0.9974 | 2.04 | 0.79 | 93.86 | 93.50 | 0.9997 |
| *S. salsa*-RP3 | 2.98 | 0.82 | 313.15 | 310.29 | 0.9996 | 1.82 | 0.67 | 108.60 | 109.34 | 0.9997 |
| *S. salsa*-RP4 | 5.25 | 0.99 | 905.86 | 916.21 | 0.9978 | 1.87 | 0.75 | 30.00 | 31.08 | 1.0000 |
| *S. salsa*-RP5 | 4.86 | 0.98 | 870.09 | 871.10 | 0.9974 | 1.60 | 0.66 | 102.57 | 105.16 | 0.9996 |
| *S. salsa*-RP6 | 4.91 | 0.98 | 573.88 | 542.45 | 0.9987 | 1.32 | 0.50 | 75.62 | 78.33 | 0.9998 |
| *S. salsa*-ES1 | 2.31 | 0.78 | 225.11 | 210.21 | 0.9993 | 2.42 | 0.82 | 30.00 | 30.38 | 1.0000 |
| *S. salsa*-ES2 | 2.63 | 0.81 | 216.30 | 215.91 | 0.9996 | 0.51 | 0.23 | 40.00 | 39.15 | 0.9998 |
| *S. salsa*-ES3 | 2.24 | 0.76 | 172.75 | 183.54 | 0.9994 | 1.55 | 0.67 | 73.50 | 73.47 | 0.9998 |
| *S. salsa*-ES4 | 2.66 | 0.82 | 347.64 | 360.58 | 0.9983 | 1.16 | 0.46 | 69.00 | 69.26 | 1.0000 |
| *S. salsa*-ES5 | 2.92 | 0.83 | 285.40 | 287.22 | 0.9996 | 0.98 | 0.53 | 16.00 | 16.00 | 1.0000 |
| *S. salsa*-ES6 | 2.10 | 0.76 | 166.00 | 190.38 | 0.9992 | 2.45 | 0.83 | 40.00 | 39.86 | 0.9999 |

**Table S2** Enriched and depleted fungal ASVs in bulk soil compare to three root-associated communities.

| OTUID | LogFC | Level | P-value | Group | Taxonomy |
| --- | --- | --- | --- | --- | --- |
| ASV001 | 6.82 | enriched | 0.000118 | *S. glauca*-BS-*S. glauca*-ES | p_Ascomycota;c_Eurotiomycetes;o_Eurotiales;f_Aspergillaceae;g_Aspergillus;g_Aspergillus |
| ASV002 | -8.76 | depleted | 0.006584 | *S. glauca*-BS-*S. glauca*-ES | p_Ascomycota;c_Dothideomycetes;o_Pleosporales;f_Pleosporaceae;g_Neocamarosporium;g_Neocamarosporium |
| ASV003 | -12.02 | depleted | 0.00043 | *S. glauca*-BS-*S. glauca*-ES | p_Ascomycota;c_Sordariomycetes;o_Xylariales;f_Diatrypaceae;g_Monosporascus;s_unidentified |
| ASV004 | -8.98 | depleted | 0.001974 | *S. glauca*-BS-*S. glauca*-ES | p_Ascomycota;c_Sordariomycetes;o_Hypocreales;f_Nectriaceae;f_Nectriaceae;f_Nectriaceae |
| ASV006 | 14.96 | enriched | 1.19E-09 | *S. glauca*-BS-*S. glauca*-ES | p_Ascomycota;c_Eurotiomycetes;o_Eurotiales;f_Aspergillaceae;g_Penicillium;s_Penicillium_glandicola |
| ASV009 | 6.10 | enriched | 0.007857 | *S. glauca*-BS-*S. glauca*-ES | p_Ascomycota;c_Sordariomycetes;o_Hypocreales;f_Nectriaceae;g_Fusarium;g_Fusarium |
| ASV017 | 8.40 | enriched | 0.001368 | *S. glauca*-BS-*S. glauca*-ES | p_Ascomycota;c_Dothideomycetes;o_Pleosporales;o_Pleosporales;o_Pleosporales;o_Pleosporales |
| ASV023 | -10.41 | depleted | 0.008841 | *S. glauca*-BS-*S. glauca*-ES | p_Ascomycota;c_Sordariomycetes;o_Hypocreales;f_Ophiocordycipitaceae;g_Purpureocillium;s_Purpureocillium_lilacinum |
| ASV051 | -12.31 | depleted | 0.007109 | *S. glauca*-BS-*S. glauca*-ES | p_Ascomycota;c_Sordariomycetes;o_Trichosphaeriales;f_Trichosphaeriaceae;g_Nigrospora;g_Nigrospora |
| ASV053 | 6.76 | enriched | 0.007182 | *S. glauca*-BS-*S. glauca*-ES | p_Ascomycota;c_Sordariomycetes;o_Hypocreales;f_Stachybotryaceae;f_Stachybotryaceae;f_Stachybotryaceae |
| ASV056 | -10.40 | depleted | 0.003949 | *S. glauca*-BS-*S. glauca*-ES | p_Ascomycota;c_Dothideomycetes;o_Pleosporales;f_Pleosporaceae;g_Exserohilum;s_Exserohilum_gedarefense |
| ASV001 | 4.74 | enriched | 0.003865 | *S. glauca*-BS-*S. glauca*-RP | p_Ascomycota;c_Eurotiomycetes;o_Eurotiales;f_Aspergillaceae;g_Aspergillus;g_Aspergillus |
| ASV006 | 5.46 | enriched | 0.000911 | *S. glauca*-BS-*S. glauca*-RP | p_Ascomycota;c_Eurotiomycetes;o_Eurotiales;f_Aspergillaceae;g_Penicillium;s_Penicillium_glandicola |
| ASV007 | 7.91 | enriched | 0.000457 | *S. glauca*-BS-*S. glauca*-RP | p_Ascomycota;c_Leotiomycetes;o_Helotiales;f_Sclerotiniaceae;g_Sclerotinia;s_Sclerotinia_sclerotiorum |
| ASV028 | -11.69 | depleted | 0.00518 | *S. glauca*-BS-*S. glauca*-RP | p_Ascomycota;c_Dothideomycetes;o_Pleosporales;f_Sporormiaceae;g_Preussia;s_Preussia_terricola |
| ASV030 | -13.57 | depleted | 0.001224 | *S. glauca*-BS-*S. glauca*-RP | p_Chytridiomycota;c_Spizellomycetes;o_Spizellomycetales;f_Powellomycetaceae;g_unidentified;s_unidentified |
| ASV040 | 7.20 | enriched | 0.00468 | *S. glauca*-BS-*S. glauca*-RP | p_Ascomycota;c_Dothideomycetes;o_Capnodiales;f_Cladosporiaceae;g_Cladosporium;g_Cladosporium |
| ASV044 | 7.38 | enriched | 0.004271 | *S. glauca*-BS-*S. glauca*-RP | p_Basidiomycota;c_Malasseziomycetes;o_Malasseziales;f_Malasseziaceae;g_Malassezia;s_Malassezia_restricta |
| ASV047 | -10.76 | depleted | 0.004184 | *S. glauca*-BS-*S. glauca*-RP | p_Ascomycota;c_Sordariomycetes;o_Hypocreales;f_Hypocreales_fam_Incertae_sedis;g_Emericellopsis;s_Emericellopsis_minima |
| ASV005 | 12.64 | enriched | 0.000453 | *S. salsa*-BS-*S. salsa*-RS | p_Ascomycota;p_Ascomycota;p_Ascomycota;p_Ascomycota;p_Ascomycota;p_Ascomycota |
| ASV011 | 6.46 | enriched | 0.004087 | *S. salsa*-BS-*S. salsa*-RS | p_Ascomycota;c_Dothideomycetes;o_Pleosporales;f_Pleosporaceae;g_Alternaria;s_Alternaria_angustiovoidea |
| ASV018 | 14.18 | enriched | 0.000108 | *S. salsa*-BS-*S. salsa*-RS | p_Ascomycota;c_Sordariomycetes;o_Microascales;f_Halosphaeriaceae;g_Corollospora;s_Corollospora_pseudopulchella |
| ASV020 | 12.68 | enriched | 0.000851 | *S. salsa*-BS-*S. salsa*-RS | p_Ascomycota;c_Sordariomycetes;o_Sordariales;f_Chaetomiaceae;g_Stolonocarpus;s_Stolonocarpus_gigasporus |
| ASV025 | 9.20 | enriched | 0.0006 | *S. salsa*-BS-*S. salsa*-RS | p_Ascomycota;c_Dothideomycetes;o_Pleosporales;f_Didymosphaeriaceae;f_Didymosphaeriaceae;f_Didymosphaeriaceae |
| ASV034 | 9.07 | enriched | 0.001528 | *S. salsa*-BS-*S. salsa*-RS | p_Ascomycota;c_Dothideomycetes;o_Pleosporales;f_Lentitheciaceae;f_Lentitheciaceae;f_Lentitheciaceae |
| ASV035 | 6.76 | enriched | 0.005493 | *S. salsa*-BS-*S. salsa*-RS | p_Ascomycota;c_Dothideomycetes;o_Pleosporales;f_Phaeosphaeriaceae;g_unidentified;s_unidentified |
| ASV043 | 9.38 | enriched | 0.001372 | *S. salsa*-BS-*S. salsa*-RS | p_Ascomycota;c_Sordariomycetes;o_Sordariales;o_Sordariales;o_Sordariales;o_Sordariales |
| ASV005 | 9.91 | enriched | 0.002552 | *S. salsa*-BS-*S. salsa*-ES | p_Ascomycota;p_Ascomycota;p_Ascomycota;p_Ascomycota;p_Ascomycota;p_Ascomycota |
| ASV006 | 13.60 | enriched | 1.23E-05 | *S. salsa*-BS-*S. salsa*-ES | p_Ascomycota;c_Eurotiomycetes;o_Eurotiales;f_Aspergillaceae;g_Penicillium;s_Penicillium_glandicola |
| ASV020 | 12.68 | enriched | 0.001905 | *S. salsa*-BS-*S. salsa*-ES | p_Ascomycota;c_Sordariomycetes;o_Sordariales;f_Chaetomiaceae;g_Stolonocarpus;s_Stolonocarpus_gigasporus |
| ASV024 | -14.19 | depleted | 0.003036 | *S. salsa*-BS-*S. salsa*-ES | p_Ascomycota;c_Eurotiomycetes;o_Eurotiales;f_Aspergillaceae;g_Aspergillus;s_Aspergillus_templicola |

**Table S3** Topological features of the co-occurrence networks of bacteria of two plants among three compartment niches.

| Network Indexes | *S. glauca*-RS  (cutoff=0.7) | *S. glauca*-RP  (cutoff=0.7) | *S. glauca*-ES  (cutoff=0.7) | *S. salsa*-RS  (cutoff=0.7) | *S. salsa*-RP  (cutoff=0.7) | *S. salsa*-ES  (cutoff=0.7) |
| --- | --- | --- | --- | --- | --- | --- |
| Total nodes | 182 | 150 | 92 | 98 | 133 | 52 |
| Total links | 2989 | 2287 | 670 | 1083 | 1626 | 198 |
| R square of power-law | 0.073 | 0.002 | 0.004 | 0.192 | 0.412 | 0.02 |
| Average degree (avgK) | 32.846 | 30.49 | 14.57 | 22.1 | 24.451 | 7.615 |
| Average clustering coefficient (avgCC) | 0.551 | 0.563 | 0.516 | 0.673 | 0.578 | 0.453 |
| Average path distance (GD) | 2.114 | 2.132 | 2.324 | 2.189 | 2.13 | 2.426 |
| Geodesic efficiency (E) | 0.542 | 0.547 | 0.502 | 0.547 | 0.54 | 0.485 |
| Harmonic geodesic distance (HD) | 1.846 | 1.83 | 1.991 | 1.828 | 1.851 | 2.06 |
| Maximal degree | 60 | 61 | 28 | 35 | 42 | 13 |
| Centralization of degree (CD) | 0.152 | 0.208 | 0.151 | 0.136 | 0.135 | 0.11 |
| Maximal betweenness | 259.897 | 240.9 | 170.9 | 230.6 | 319.172 | 145.347 |
| Centralization of betweenness (CB) | 0.01 | 0.014 | 0.027 | 0.038 | 0.029 | 0.087 |
| Maximal stress centrality | 7146 | 6133 | 1695 | 3478 | 4901 | 617 |
| Centralization of stress centrality (CS) | 0.238 | 0.353 | 0.256 | 0.535 | 0.407 | 0.365 |
| Maximal eigenvector centrality | 0.163 | 0.157 | 0.228 | 0.189 | 0.184 | 0.291 |
| Centralization of eigenvector centrality (CE) | 0.106 | 0.098 | 0.153 | 0.117 | 0.119 | 0.176 |
| Density (D) | 0.181 | 0.205 | 0.16 | 0.228 | 0.185 | 0.149 |
| Transitivity (Trans) | 0.567 | 0.634 | 0.551 | 0.688 | 0.591 | 0.443 |
| Efficiency | 0.823 | 0.801 | 0.849 | 0.78 | 0.821 | 0.867 |

**Table S4** Topological features of the co-occurrence networks of bacteria and fungi of two plants among three compartment niches.

| Network Indexes | *S. glauca*-RS  (cutoff=0.7) | *S. glauca*-RP  (cutoff=0.7) | *S. glauca*-ES  (cutoff=0.7) | *S. salsa*-RS  (cutoff=0.7) | *S. salsa*-RP  (cutoff=0.7) | *S. salsa*-ES  (cutoff=0.7) |
| --- | --- | --- | --- | --- | --- | --- |
| Total nodes | 200 | 207 | 115 | 111 | 163 | 71 |
| Total links | 3308 | 3648 | 969 | 1214 | 2239 | 387 |
| R square of power-law | 0.071 | 0.019 | 0.232 | 0.021 | 0.237 | 0.094 |
| Average degree (avgK) | 33.08 | 35.25 | 16.85 | 21.87 | 27.47 | 10.9 |
| Average clustering coefficient (avgCC) | 0.523 | 0.537 | 0.507 | 0.63 | 0.559 | 0.45 |
| Average path distance (GD) | 2.129 | 2.15 | 2.306 | 2.245 | 2.157 | 2.284 |
| Geodesic efficiency (E) | 0.534 | 0.532 | 0.5 | 0.528 | 0.531 | 0.507 |
| Harmonic geodesic distance (HD) | 1.873 | 1.88 | 2.001 | 1.893 | 1.885 | 1.974 |
| Maximal degree | 60 | 65 | 30 | 37 | 46 | 18 |
| Centralization of degree (CD) | 0.137 | 0.146 | 0.117 | 0.14 | 0.116 | 0.104 |
| Maximal betweenness | 280.849 | 392 | 188.7 | 235.1 | 323.3 | 124.4 |
| Centralization of betweenness (CB) | 0.009 | 0.013 | 0.018 | 0.028 | 0.018 | 0.033 |
| Maximal stress centrality | 8306 | 13587 | 2900 | 3954 | 7067 | 757 |
| Centralization of stress centrality (CS) | 0.239 | 0.441 | 0.302 | 0.456 | 0.386 | 0.198 |
| Maximal eigenvector centrality | 0.161 | 0.154 | 0.208 | 0.184 | 0.168 | 0.241 |
| Centralization of eigenvector centrality (CE) | 0.107 | 0.104 | 0.137 | 0.116 | 0.109 | 0.145 |
| Density (D) | 0.166 | 0.171 | 0.148 | 0.199 | 0.17 | 0.156 |
| Transitivity (Trans) | 0.547 | 0.572 | 0.526 | 0.662 | 0.576 | 0.465 |
| Efficiency | 0.838 | 0.833 | 0.86 | 0.808 | 0.836 | 0.856 |

**Table S5** The keystone species distribution of microorganisms of two plant species.

| ID | Taxonomy | Node degree | Node between |
| --- | --- | --- | --- |
| *S. glauca*-RS-1 | d_Bacteria;p_Bacteroidota;c_Bacteroidia;o_Flavobacteriales;f_Flavobacteriaceae;g_Flavobacterium | 48 | 90.488867 |
| *S. glauca*-RS-2 | d_Bacteria;p_Myxococcota;c_Polyangia;o_Polyangiales;f_Sandaracinaceae;g_uncultured | 48 | 90.488867 |
| *S. glauca*-RS-3 | d_Bacteria;p_Proteobacteria;c_Gammaproteobacteria;o_Xanthomonadales;f_Rhodanobacteraceae; | 48 | 90.488867 |
| *S. glauca*-RS-4 | d_Bacteria;p_Actinobacteriota;c_Acidimicrobiia;o_Actinomarinales;f_uncultured;g_uncultured | 49 | 101.83378 |
| *S. glauca*-RS-5 | d_Bacteria;p_Proteobacteria;c_Gammaproteobacteria;o_Burkholderiales;f_Comamonadaceae; | 49 | 101.83378 |
| *S. glauca*-RS-6 | d_Bacteria;p_Proteobacteria;c_Gammaproteobacteria;o_Pseudomonadales;f_Cellvibrionaceae;g_Cellvibrio | 49 | 101.83378 |
| *S. glauca*-RS-7 | d_Bacteria;p_Proteobacteria;c_Gammaproteobacteria;o_Pseudomonadales;f_Halomonadaceae;g_Halomonas | 53 | 108.97696 |
| *S. glauca*-RS-8 | d_Bacteria;p_Proteobacteria;c_Gammaproteobacteria;o_Burkholderiales;f_Oxalobacteraceae;g_Massilia | 56 | 109.31856 |
| *S. glauca*-RP-1 | d_Bacteria;p_Proteobacteria;c_Gammaproteobacteria;o_Acidithiobacillales;f_Acidithiobacillaceae;g_RCP1-48 | 55 | 72.755544 |
| *S. glauca*-RP-2 | d_Bacteria;p_Proteobacteria;c_Alphaproteobacteria;o_Rhizobiales | 50 | 89.478909 |
| *S. glauca*-RP-3 | d_Bacteria;p_Bacteroidota;c_Bacteroidia;o_Flavobacteriales;f_Flavobacteriaceae;g_Flavobacterium | 51 | 94.281243 |
| *S. glauca*-RP-4 | d_Bacteria;p_Proteobacteria;c_Gammaproteobacteria;o_Pseudomonadales;f_Pseudomonadaceae;g_Pseudomonas | 57 | 106.50836 |
| *S. glauca*-RP-5 | d_Bacteria;p_Gemmatimonadota;c_PAUC43f_MBG;o_PAUC43f_MBG;f_PAUC43f_MBG;g_PAUC43f_MBG | 57 | 106.50836 |
| *S. glauca*-RP-6 | d_Bacteria;p_Bacteroidota;c_Bacteroidia;o_Flavobacteriales;f_Flavobacteriaceae;g_Ulvibacter | 57 | 106.50836 |
| *S. glauca*-RP-7 | d_Bacteria;p_Actinobacteriota;c_Actinobacteria;o_Micrococcales;f_Micrococcaceae;g_Kocuria | 57 | 106.50836 |
| *S. glauca*-RP-8 | d_Bacteria;p_Proteobacteria;c_Alphaproteobacteria;o_Rhizobiales;f_Beijerinckiaceae;g_Microvirga | 57 | 108.79071 |
| *S. glauca*-ES-1 | d_Bacteria;p_Proteobacteria;c_Alphaproteobacteria;o_Rhodobacterales;f_Rhodobacteraceae;g_Defluviimonas | 24 | 49.353971 |
| *S. glauca*-ES-2 | d_Bacteria;p_Proteobacteria;c_Alphaproteobacteria;o_Rhizobiales;f_Methyloligellaceae;g_Methyloceanibacter | 25 | 49.364872 |
| *S. glauca*-ES-3 | d_Bacteria;p_Bacteroidota;c_Bacteroidia;o_Cytophagales;f_Microscillaceae;g_uncultured | 28 | 50.519114 |
| *S. glauca*-ES-4 | d_Bacteria;p_Proteobacteria;c_Gammaproteobacteria;o_Pseudomonadales;; | 28 | 50.519114 |
| *S. glauca*-ES-5 | d_Bacteria;p_Proteobacteria;c_Gammaproteobacteria;o_Nitrosococcales;f_Methylophagaceae;g_Methylophaga | 23 | 58.581322 |
| *S. salsa*-RS-1 | d_Bacteria;p_Firmicutes;c_Bacilli;o_Bacillales;f_Bacillaceae;g_Bacillus | 33 | 19.682195 |
| *S. salsa*-RS-2 | d_Bacteria;p_Proteobacteria;c_Alphaproteobacteria;o_Rhizobiales;f_Stappiaceae;g_Labrenzia | 33 | 19.682195 |
| *S. salsa*-RS-3 | d_Bacteria;p_Bacteroidota;c_Bacteroidia;o_Cytophagales;f_Cyclobacteriaceae;g_Marinoscillum | 33 | 19.682195 |
| *S. salsa*-RS-4 | d_Bacteria;p_Proteobacteria;c_Alphaproteobacteria;o_Rhodobacterales;f_Rhodobacteraceae;g_Paracoccus | 33 | 19.682195 |
| *S. salsa*-RS-5 | d_Bacteria;p_Proteobacteria;c_Gammaproteobacteria___;;; | 33 | 19.682195 |
| *S. salsa*-RP-1 | d_Bacteria;p_Proteobacteria;c_Gammaproteobacteria;o_Granulosicoccales;f_Granulosicoccales;g_Methylohalomonas | 37 | 32.190913 |
| *S. salsa*-RP-2 | d_Bacteria;p_Proteobacteria;c_Alphaproteobacteria;o_Rhodobacterales;f_Rhodobacteraceae;g_uncultured | 40 | 40.67553 |
| *S. salsa*-RP-3 | d_Bacteria;p_Actinobacteriota;c_Acidimicrobiia;o_Microtrichales;f_uncultured;g_uncultured | 40 | 40.67553 |
| *S. salsa*-RP-4 | d_Bacteria;p_Proteobacteria;c_Alphaproteobacteria;o_Kiloniellales;f_Kiloniellaceae;g_Tistlia | 40 | 40.67553 |
| *S. salsa*-RP-5 | d_Bacteria;p_Myxococcota;c_Polyangia;o_Haliangiales;f_Haliangiaceae;g_Haliangium | 37 | 49.241672 |
| *S. salsa*-RP-6 | d_Bacteria;p_Gemmatimonadota;c_BD2-11_terrestrial_group;o_BD2-11_terrestrial_group;f_BD2-11_terrestrial_group;g_BD2-11_terrestrial_group | 39 | 57.242349 |
| *S. salsa*-RP-7 | d_Bacteria;p_Verrucomicrobiota;c_Verrucomicrobiae;o_Pedosphaerales;f_Pedosphaeraceae;g_Pedosphaeraceae | 39 | 57.242349 |
| *S. salsa*-RP-8 | d_Bacteria;p_Myxococcota;c_Polyangia;o_Polyangiales;f_BIrii41;g_BIrii41 | 39 | 57.242349 |
| *S. salsa*-ES-1 | k_Fungi;p_Ascomycota;c_Saccharomycetes;o_Saccharomycetales;f_Saccharomycetales_fam_Incertae_sedis;g_Candida | 14 | 38.478571 |
| *S. salsa*-ES-2 | d_Bacteria;p_Proteobacteria;c_Gammaproteobacteria;o_Pseudomonadales;f_Marinobacteraceae;g_Marinobacter | 15 | 40.053017 |
| *S. salsa*-ES-3 | d_Bacteria;p_Proteobacteria;c_Alphaproteobacteria;o_Sphingomonadales;f_Sphingomonadaceae;g_Sphingomonas | 14 | 45.133228 |
| *S. salsa*-ES-4 | k_Fungi;p_Mortierellomycota;c_Mortierellomycetes;o_Mortierellales;f_Mortierellaceae;g_Mortierella | 14 | 46.818662 |


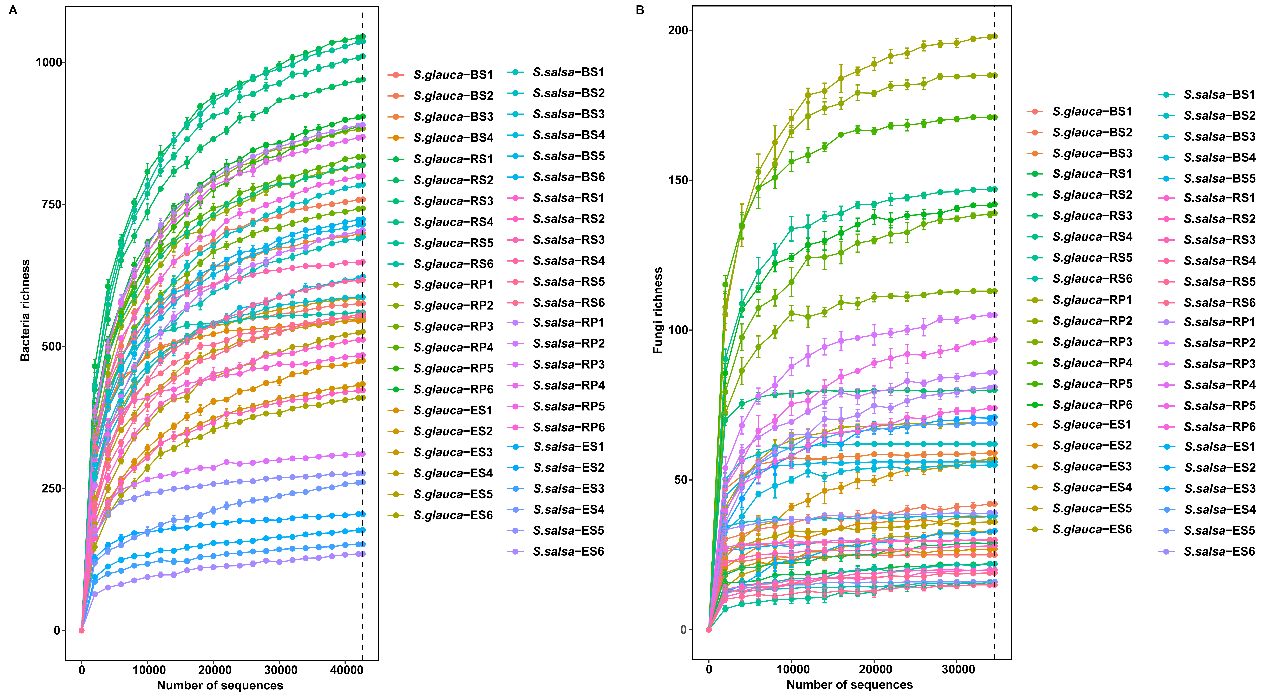


**Fig. S1** Species accumulation curve based on the ASV level bacterial (A) and fungal (B) community.


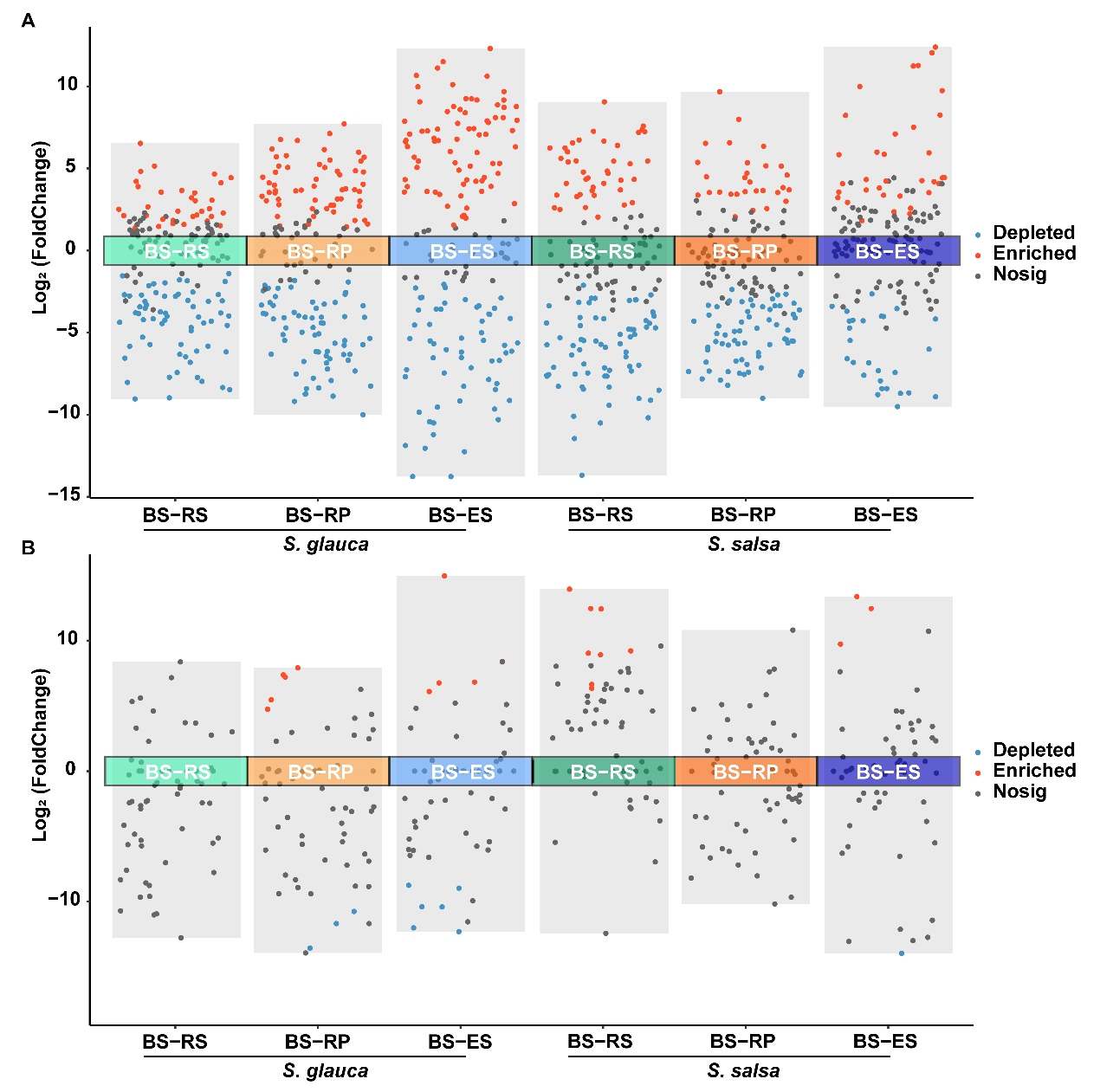


**Fig. S2** The enriched and depleted genera in RS, RP, and ES compared with BS in bacterial (A) and fungal (B) community. BS, bulk soil; RS, rhizosphere; RP, rhizoplane; ES, endosphere.


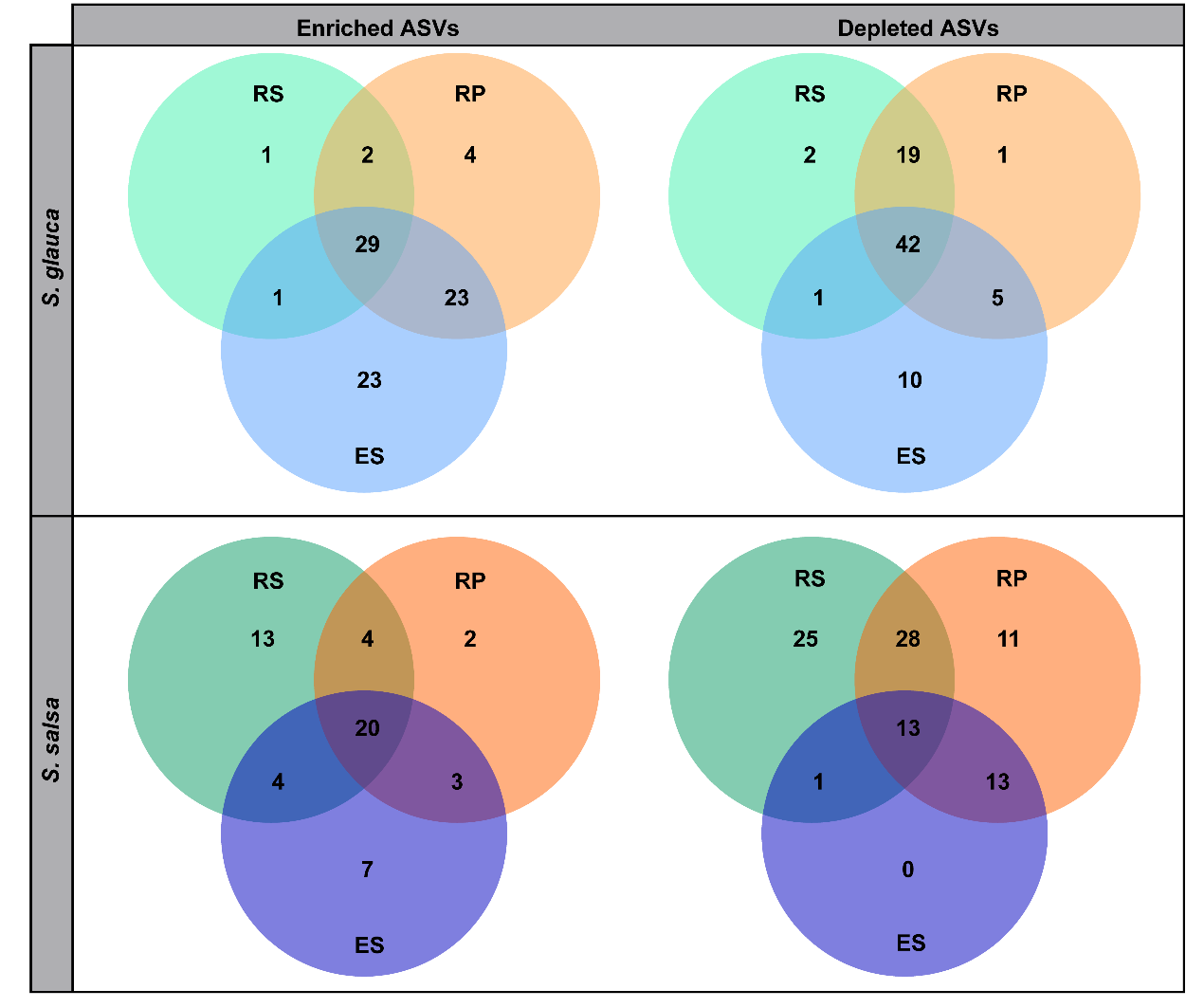


**Fig. S3** Venn diagram showing the number of enriched or deplete ASVs in each compartment of two plants. BS, bulk soil; RS, rhizosphere; RP, rhizoplane; ES, endosphere.


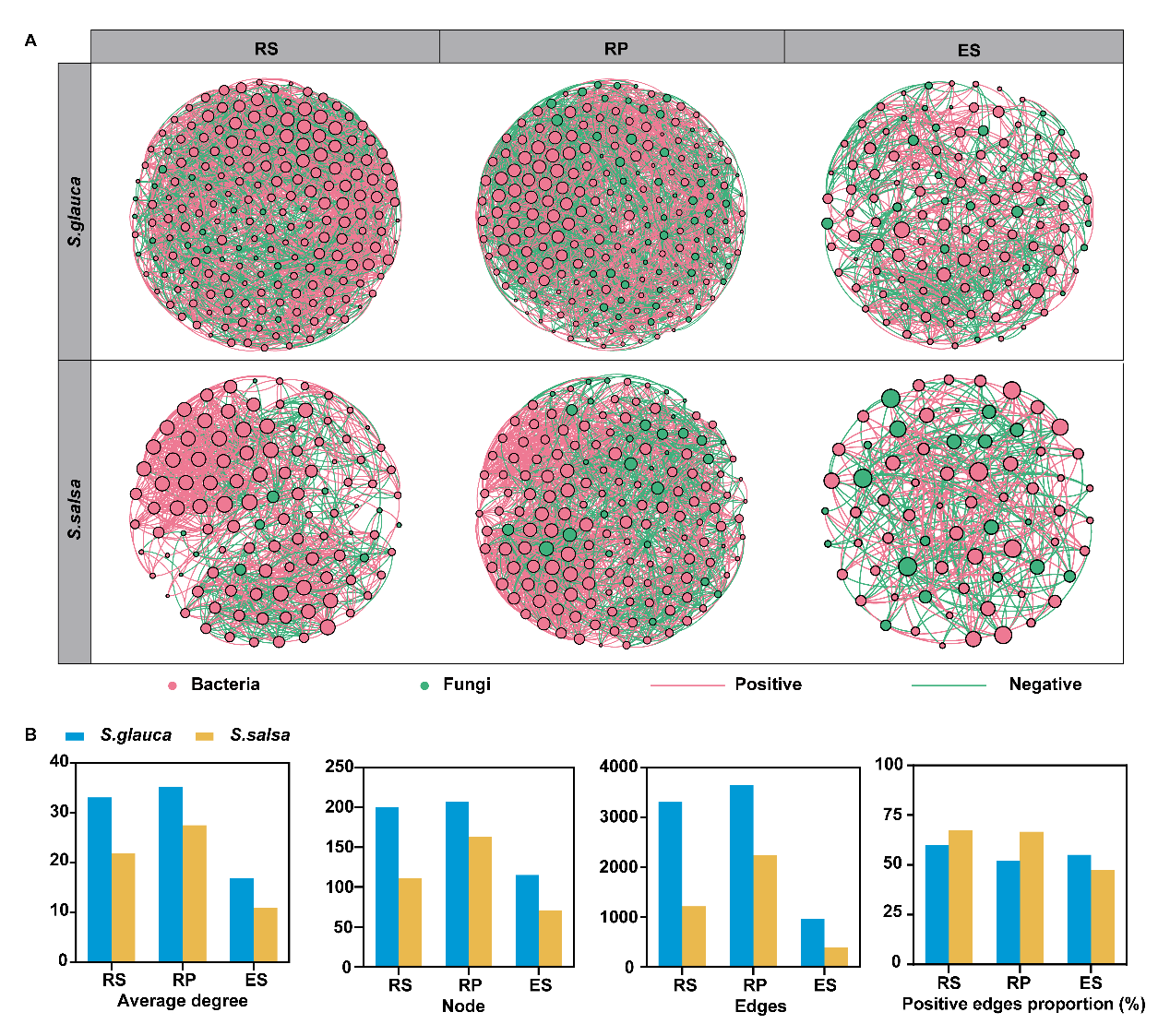


**Fig. S4** Pattens of the microbial co-occurrence networks between two plants across three root-associated compartments. A, Co-occurrence network analysis of diseased and healthy plants along the soil-root continuum. Nodes represent dominant bacterial and fungal genus (with relative abundances > 0.1%) that were shown in different colors based on the taxonomy of domain. The size of each node is proportional to the degree of genus. Lines in red and green denote positive and negative correlations, respectively. B, Network topological parameters for three niches microbial networks.


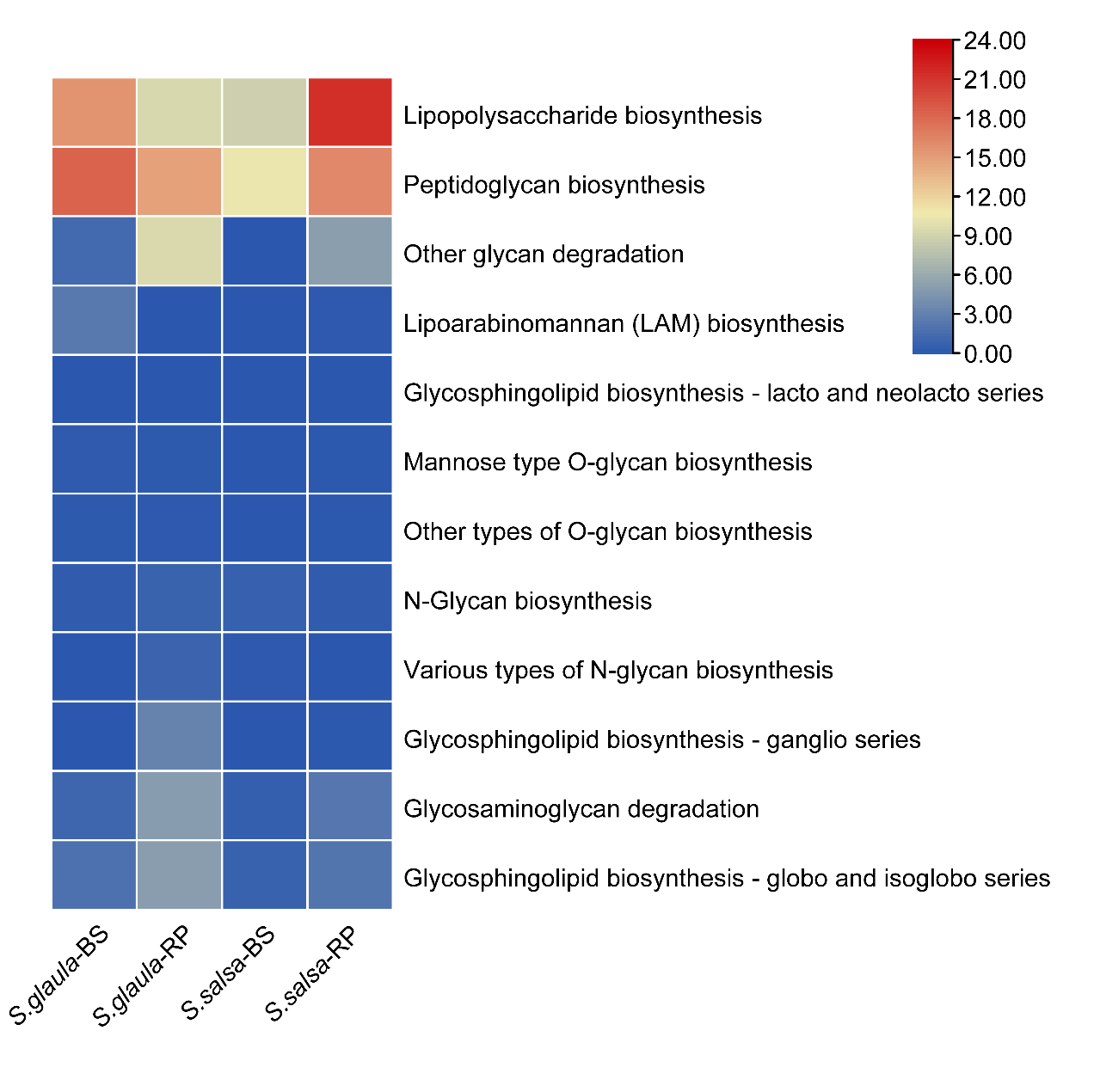


**Fig. S5** Heatmap showing the KEGG orthologue group (KO) affiliated with glycan biosynthesis and metabolism.
